# The trajectory of vesicular proteomic signatures from HBV‐HCC by chitosan‐magnetic bead‐based separation and DIA‐proteomic analysis

**DOI:** 10.1002/jev2.12499

**Published:** 2024-08-29

**Authors:** Lin Cao, Yue Zhou, Shuai Lin, Chunyan Yang, Zixuan Guan, Xiaofan Li, Shujie Yang, Tong Gao, Jiazhen Zhao, Ning Fan, Yanan Song, Dongmin Li, Xiang Li, Zhuo Li, Feng Guan, Zengqi Tan

**Affiliations:** ^1^ Institute of Hematology Provincial Key Laboratory of Biotechnology, School of Medicine Northwest University Xi'an Shaanxi China; ^2^ Key Laboratory of Resource Biology and Biotechnology in Western China, Ministry of Education, Provincial Key Laboratory of Biotechnology, College of Life Sciences Northwest University Xi'an Shaanxi China; ^3^ Department of Oncology The Second Affiliated Hospital of Xi'an Jiaotong University Xi'an Shaanxi China; ^4^ Institute of Basic and Translational Medicine Xi'an Medical University Xi'an Shaanxi China; ^5^ Department of Biochemistry and Molecular Biology, School of Basic Medical Sciences Xi'an Jiaotong University Health Science Center Xi'an Shaanxi P.R. China; ^6^ Department of Laboratory Medicine The First Affiliated Hospital of Xi'an Medical University Xi'an Shaanxi P.R. China

**Keywords:** hepatocellular carcinoma, proteomics, small extracellular vesicles, polysaccharide chitosan

## Abstract

Hepatocellular carcinoma (HCC) is a prevalent primary liver cancer often associated with chronic hepatitis B virus infection (CHB) and liver cirrhosis (LC), underscoring the critical need for biomarker discovery to improve patient outcomes. Emerging as a promising avenue for biomarker development, proteomic technology leveraging liquid biopsy from small extracellular vesicles (sEV) offers new insights. Here, we evaluated various methods for sEV isolation and identified polysaccharide chitosan (CS) as an optimal approach. Subsequently, we employed optimized CS‐based magnetic beads (Mag‐CS) for sEV separation from serum samples of healthy controls, CHB, LC, and HBV‐HCC patients. Leveraging data‐independent acquisition mass spectrometry coupled with machine learning, we uncovered potential vesicular protein biomarker signatures (KNG1, F11, KLKB1, CAPNS1, CDH1, CPN2, NME2) capable of distinguishing HBV‐HCC from CHB, LC, and non‐HCC conditions. Collectively, our findings highlight the utility of Mag‐CS‐based sEV isolation for identifying early detection biomarkers in HBV‐HCC.

## 1 INTRODUCTION

Primary liver cancer is the sixth most commonly diagnosed cancer and the third leading cause of cancer deaths worldwide in 2020, with 905,677 newly diagnosed cases and 830,180 new death cases (Sung et al., 2021). Hepatocellular carcinoma (HCC) constitutes 75%–85% of the primary liver cancer (Sung et al., 2021). Among the risk factors for HCC, chronic hepatitis B virus infection (CHB) predominates, primarily due to the high prevalence of HBV infection in China (London et al., 2006; Yue et al., 2022). CHB and the subsequent continuous chronic inflammation are strongly associated with the development of fibrosis, cirrhosis and HCC (Fattovich et al., 2004; Luedde & Schwabe, 2011). Despite advancements in therapeutic options for HCC, the overall prognosis remains discouraging with a 5‐year survival rate below 20% (Jemal et al., 2017). Alpha‐fetoprotein (AFP) is the most widely used biomarker for HCC. Nevertheless, the specificity and sensitivity of AFP are relatively limited (Bruix & Sherman, 2011; Marrero et al., 2009), and elevated AFP levels can be detected in non‐HCC malignancies and other chronic liver inflammations (Befeler & Di Bisceglie, 2002; Johnson, 2001). Therefore, there is an ongoing need to improve HCC surveillance, particularly for HBV‐associated HCC (HBV‐HCC), to facilitate early detection.

Given the considerable limitations of traditional solid biopsy, it is imperative to introduce liquid biopsy into clinical practice for less invasive operation and early detection. Among liquid biopsy analytes, small extracellular vesicles (sEV) have emerged as a platform with broader and complementary applications (Zhao et al., 2022). sEV, a type of membrane‐enclosed nanoparticles with a diameter of 30–150 nm, is released by almost all cell types into the surrounding biofluids, playing critical roles in various physiological and pathological process (Mathieu et al., 2019). sEV encloses a variable spectrum of molecules that reflect their parent cells, including nucleic acids, proteins and lipids (Yanez‐Mo et al., 2015). The remarkable stability of sEV ensures the integrity of molecules cargos. Moreover, sEV is present in the circulation at relatively early stages of diseases, and sustains across all disease processes. Thus, sEV provides broad opportunities for clinically relevant diagnostics. For example, an HCC sEV‐derived 10‐gene molecular signature exhibits a high sensitivity of 94.4% and specificity of 88.5% for early HCC detection (Sun et al., 2020). However, the trajectories of circulating vesicular proteins in the HCC progression and vesicular protein signatures remain relatively understudied.

The efficient enrichment of sEV from bodily fluids is a critical procedure for subsequent molecular analysis and clinically relevant diagnostics. sEV separation methods are primarily based on their physical properties or composition (Macias et al., 2018), and sEV quality is significantly influenced by the sEV separation procedure (Brennan et al., 2020). Commonly, sEV separation methods are broadly categorized into differential ultracentrifugation (UC), density gradient centrifugation (GDC), size exclusion chromatography (SEC), immuno‐capture (IC), polyethylene glycol (PEG) precipitation, microfluidic approaches, etc. (Mateescu et al., 2017). In addition to these classic separation methods, novel sEV separation methods have been documented (Petga et al., 2024). Recently, the polysaccharide chitosan (CS) has been employed for sEV separation by the interaction between the high positive charge of CS and the negatively‐charged membranes of sEV (Kumar et al., 2021). It is crucial to assess and identify an optimal method for clinically relevant sEV diagnostic.

In this work, sEV was first separated from serum employing UC, GDC, IC SEC, PEG and CS, and evaluated using western blot, transmission electron microscopy, nanoparticle tracking analysis, and mass spectrometry. Magnetic beads prepared with diverse Fe_3_O_4_ nanoparticle sizes, coated with differently deacetylated CS were further compared for sEV enrichment. In addition, sEV derived from serum samples from HC, CHB, LC, and HBV‐HCC patients were separated using CS‐coated magnetic beads, and subjected to data‐independent acquisition mass spectrometry (DIA‐MS) and cancerous trajectory analysis. Finally, vesicular biomarker signatures specific to HBV‐HCC were identified by employing machine learning.

## 2 MATERIALS AND METHODS

### 2.1 Serum sample collection and pretreatment

A total of 192 serum samples in the training cohort (HC = 20, CHB = 24, LC = 23, HCC = 29) and validation cohort (HC = 18, CHB = 26, LC = 26, HCC = 26) were collected from the First Affiliated Hospital of Xi'an Medical University and the Second Affiliated Hospital of Xi'an Jiaotong University (Table S1). All blood donors have signed informed consent, in accordance with the Declaration of Helsinki guidelines. The serum was centrifuged at 2000×*g* for 30 min. The supernatant was collected, centrifuged at 12,000×*g* for 45 min at 4°C, filtered through a 0.22 μm pore filter (Sigma–Aldrich, Saint Louis, USA), and collected for further sEV extraction or stored at −80°C. An equal volume of serum (200 μL) was utilized for sEV separation for subsequent proteomic analysis or western blot. Pooled serum was used as quality control, that was included within the sample cohorts in proteomics.

### 2.2 UC

Preprocessed serum was ultra‐centrifuged at 110,000×*g* for 70 min at 4°C (Optima XE‐100; Beckman coulter life Sciences; Indianapolis, IN, USA). sEV pellets were rinsed with PBS, collected by UC at 110,000×*g* for 70 min, resuspended in PBS, and stored at −80°C.

### 2.3 DGC

sEV was purified by DGC as previously described (Sung et al., 2015). Briefly, 40%, 20%, 10% and 5% (w/v) iodixanol solutions were prepared by diluting a stock solution (60%, w/v) of OptiPrep (Axis‐Shield PoC, AS, Oslo, Norway) with 0.25 M sucrose/ 10mM Tris, pH 7.5. A discontinuous iodixanol gradient was added to the ultra‐clear centrifuge tube from bottom to top, and sEV pre‐separated by UC was added on the top of gradient. The tube was then centrifuged at 100,000×*g* for 18 h at 4°C. Twelve individual 1 mL gradient fractions were collected. Each fraction was diluted with PBS and centrifuged at 110,000×*g* for 70 min at 4°C. The resulting pellets were resuspended in PBS.

### 2.4 SEC

qEV original 70 columns (IZON Science, Christchurch, New Zealand) were used for sEV separation according to the manufacturer. Briefly, the columns were equilibrated with PBS. Pretreated serum sample was loaded to the column, followed by elution with PBS. sEV‐enriched fractions (Befeler & Di Bisceglie, 2002; Bruix & Sherman, 2011; Marrero et al., 2009) were pooled and stored at −80°C.

### 2.5 PEG precipitation

sEV was separated by PEG precipitation as previously described (Rider et al., 2016). In brief, an equal volume of PEG stock solution, containing 1.6 g/L PEG 6000 and 1 M NaCl, was added to the pretreated serum. The solution was incubated overnight at 4°C with gentle agitation, and centrifugated at 16,400×*g* for 1 h at 4°C. The pellets were resuspended in PBS.

### 2.6 Immunoaffinity capture (IC)

Immunoaffinity capture was performed as previously described (Jeppesen et al., 2019; Zarovni et al., 2015). The pretreated serum was incubated with magnetic beads directly conjugated to antibodies against CD63, CD81 or CD9 (Invitrogen, Carlsbad, CA, USA) for 16 h at 4°C on a rotating mixer. Subsequently, the beads were rinsed four times with 0.1% BSA‐PBS, and rinsed one time with PBS. Finally, sEV was eluted with carbonate‐bicarbonate solution (pH 11.3) (Multia et al., 2019) from the magnetic beads by vibrating for 10 min.

For proteomic analysis, sEV were lysed and denatured with 8 M urea and 50 mM NH_4_HCO_3_. For intact sEV release, sEV was further neutralized immediately with 1 M HCl, and replaced with PBS through 10 kDa ultrafiltration tube (Millipore).

### 2.7 CS‐based isolation

CS precipitation was performed as previously described (Huang et al., 1984). In brief, CS was dissolved in 1% (w/w) acetic acid at a concentration of 20 mg/mL, and CS (final concentration 50 μg/mL) was added to preprocessed serum, incubated for 1 h at room temperature (RT) with end‐to‐end rotation, and centrifuged at 12,000×*g* for 15 min at 4°C. The pellets were rinsed three times with PBS and centrifuged at 12,000×*g* for 10 min at 4°C.

For proteomic analysis, the pellets were lysed and denatured with 8 M urea and 50 mM NH_4_HCO_3_. For intact sEV release, the pellets were added with 2 M NaCl, vigorously vortexed for 5 min, and incubated overnight with shaking at RT, followed by centrifugation at 17,000×*g* for 15 min at 4°C. The supernatant containing sEV was collected and replaced with PBS with a 10 kDa ultrafiltration tube.

### 2.8 Transmission electron microscopy (TEM)

sEV separated by each method was deposited onto the carbon film covered 200 mesh copper grids for 2 min. The excess solution was soaked off by a filter paper. The samples were negatively stained with 2% uranyl acetate for 30 s, and the excess stain was soaked off. The grids were visualized by TEM (H‐7650; Hitachi, Tokyo, Japan) at 80 kV.

### 2.9 Dynamic light scattering

sEV was loaded into a NanoSight LM10 instrument (Malvern; UK), and particles were tracked for 60 s using the NanoSight nanoparticle tracking analysis software program.

### 2.10 Western blotting

sEV proteins were loaded and separated by SDS/PAGE, and transferred onto PVDF membrane (Bio‐Rad; Hercules, CA, USA). Membranes were blocked with 3% BSA in TBST (Tris buffered saline with Tween) for 1 h at RT, incubated with primary antibodies against APOB (20578‐1‐AP; Proteintech, Wuhan, China), CD63 (A19023; ABclonal, Wuhan, China), TSG101 (A1692; ABclonal) and ALIX (A2215; ABclonal) overnight at 4°C, probed with the HRP‐conjugated secondary antibody (Beyotime) for 1 h at RT. The bands were visualized by chemiluminescence reagents.

### 2.11 Preparation of CS‐coated magnetic beads

CS‐coated magnetic beads (Mag‐CS) were prepared as previously described (He et al., 2010). In brief, Fe_3_O_4_ nanoparticles (0.2 g) dispersed in a solution with 30 mL paraffin and 0.5 mL span‐80 were added with 7 mg/mL CS in 2% (w/w) acetic acid, and mixed by ultrasonic irradiation for 30 min, and further mixed with 3 mL 25% glutaraldehyde solution for 4 h by a mechanical stirrer. The Mag‐CS were rinsed with H_2_O and ethanol, and dried at 50°C.

Mag‐CS were added to preprocessed serum at a concentration of 2 mg/mL, incubated for 1 h at RT by end‐to‐end rotation, rinsed with PBS, and collected with a magnetic stand. For proteomic analysis, the sEV were denatured with 8 M urea and 50 mM NH_4_HCO_3_, and digested with trypsin on beads. For intact sEV release, the beads were incubated with 2 M NaCl, and the supernatant containing sEV was collected and replaced with PBS with a 10 kDa ultrafiltration tube.

### 2.12 Determination of deacetylation degree of CS

Deacetylation degree of CS was determined as previously described (Yang et al., 2022). In brief, CS (0.2 g) was dissolved in 30 mL of 0.1 mol/L HCl aqueous solution. Using methyl orange as an indicator, the excess HCl was titrated with 0.1 M NaOH to the end point. The degree of deacetylation of CS was determined according to the following formula.

DD=(C1V1−C2V2)×0.016/G(100%−W)×0.0994×100%

where C1, C2 are the concentration of HCl and NaOH (mol/L), respectively; V1, V2 represent the volume of dropped HCl and consumed NaOH (mL), respectively; G: sample weight (g); W: sample moisture content (%); 0.016: amount of amine equivalent to 1 mL of 1 mol/L HCl (g); 0.094: theoretical amino group content in CS.

### 2.13 DIA‐MS

Proteins were digested and desalted as previously described (Tan et al., 2020). In brief, sEV proteins of an equal volume of serum (200 μL) were denatured in 8 M urea and 50 mM NH_4_HCO_3_, reduced with DTT, alkylated with IAM, digested with lysyl endopeptidase (Promega; Madison, WI, USA) for 4 h at 37°C, and then incubated with trypsin (Promega) overnight at 37°C with shaking. The digested peptides were acidified with 10% trifluoroacetic acid to pH < 3, collected by centrifugation, desalted using Oasis HLB cartridges (Waters; Milford, MA, USA).

Peptides were resuspended in 20 μL 0.1% formic acid (FA) in water (A) and peptides (2 μL) were analysed using Q Exactive HF‐X (Thermo Fisher) equipped with UltiMate 3000 RSLCnano (Thermo Fisher). Peptides were eluted from an analytical column (Acclaim PepMap C18, 2 μm, 100 Å, 75 μm × 25 cm; Thermo Fisher) using a 90‐min gradient of 1%–8% solution B (80% ACN containing 01% FA) for 4 min, 8%–30% B for 76 min, 30%–90% B for 6 min and 100% B for 4 min with flow rate of 0.4 μL/min. The MS was operated under DIA mode using 60 DIA segments with an isolation window of 10 Th covering *m*/*z* 400–1000. The isolation lists were 400–410, 410–420, 420–430, 430–440 and so forth.

DIA data were analysed using DIA‐NN (v1.8.0) with default settings (Precursor FDR: 1%, Log lev: 1, Mass accuracy: 0, MS1 accuracy: 0, Scan window: 0, Protein interference: genes, Quantification strategy: robust LC (high precision), Neural network classification: single‐pass mode, MBR: on). Library‐free search strategy was used where human spectral library was predicated with settings (Deep learning: on, Protease: Trypsin/P, Missed cleavage: 1, N‐term M excision: on, C carbmidomethylation: on, Peptide length range: 7–30, Precursor charge range: 2–4, Precursor *m*/*z* range: 400–1000, Fragment ion *m*/*z* range: 200–1800).

### 2.14 Proteomic analysis

Proteins identified as ‘reverse’ and ‘contaminants’ were removed for further analysis. In Perseus software (version 2.0.6.0) (Tyanova et al., 2016), proteins with at least 70 percentage valid values in each group were considered and used for further analysis. Intensities were log2‐transformed and normalized, and missing values were replaced with the width of the Gaussian distribution set to 0.3 and a down shift of 1.8 using Perseus. Differentially expressed proteins were compared in HBV‐HCC compared to HC, CHB and LC using cut‐offs of fold change >1.2, fold change <0.83, and *p*‐value < 0.05.

The PLS‐DA were performed using mixOmics (version 6.22.0) and ggplot2 (version 3.4.2) packages using R (version 4.2.3). Hierarchical clustering was performed using pheatmap package (version 1.0.12). Gene ontology (GO) enrichment analysis was performed using g:Profiler (Kolberg et al., 2023). Protein sets, Vesiclepedia top 100 list (version 5.1) was downloaded from Vesiclepedia database (Pathan et al., 2019). Vesicle‐related GO list, Proteins present in the Cellular Compartment GO terms, and blood protein list were based on a previous study (Veerman et al., 2021).

### 2.15 Machine learning

The protein intensity data, which had been Log2‐transformed, were Z‐scored within two cohorts. Cohort 1 was used as the training set to develop the prediction model, while Cohort 2 served as the validation set to evaluate the trained classifier. We applied 5‐fold cross‐validation on the training data to derive the HCC predictive model based on binary classification comparisons: HCC versus non‐HCC, HCC versus CHB, and HCC versus LC.

### 2.16 Feature selection

To identify protein biomarkers that discriminated between HCC versus Non‐HCC, HCC versus CHB, and HCC versus LC, differentially expressed proteins which were directionally concerted in HBV‐HCC compared to HC, CHB and LC, and annotated as vesicular proteins in both Vesiclepedia and Exocarta dataset, were selected for feature selection. We utilized six machine learning classifier methods (Logistic Regression, Linear SVM, RBF SVM, Decision Tree, Random Forest, and AdaBoost) and the Sequential Feature Selection (SFS) method available in scikit‐learn (version 1.4.2) using Python 3.10 (https://www.python.org/). The SFS method was implemented in the ‘sklearn.feature_selection.SequentialFeatureSelector’ transformer. We opted for Forward‐SFS, which was a greedy procedure that iteratively identifies the best new feature to add to the set of selected features, fitting on the training set.

The hyperparameters of SFS were evaluated through various iterative combinations: estimator was one of six machine learning classifier methods; n_features_to_select ranging from 1 to 14 with an interval of 1; direction = 'forward'; scoring = 'roc‐auc'; and the others hyperparameters were default values. The hyperparameters of each classifier model were optimized by grid search using sklearn.model_selection.GridSearchCV function. For a specific machine learning method, we selected the top 10 protein combinations with the highest metric scores in each iteration. These top combinations were then combined to form candidate feature sets used for downstream analysis, that is, model evaluation.

### 2.17 Model performance evaluation

Validation set was used to evaluate on selected predictive models. The ROC (receiver operating characteristic) curve, which illustrates the relationship between the TPR (true positive rate) and FPR (false positive rate), was calculated for both training and validation set. To better get insight into the performance of imbalanced models, PRC (precision‐recall curve) was also showed representation of the trade‐off between precision and recall. ROC was visualized for models using matplotlib (version 3.8.0) and seaborn (version 0.13.2) package on three comparisons. The AUROC (area under the ROC curve) and AP (average precision for PRC) were calculated using sklearn.metrics function. To assess the sensitivity and specificity and NPV of the final model with 0.5 cutoff value, we calculated the elements in the confusion matrix using sklearn.metrics. Bootstrap method was applied to estimate 95% confidence interval (CI) for model metrics, such as the AUROC, AP, sensitivity, specificity, and NPV. Shapley additive explanations method using SHAP (version 0.45.0) package was used to estimate the impact of features on the three HCC predictive models.

### 2.18 Statistical analysis

All statistical analyses were performed using the GraphPad Prism V.7.0 software program GraphPad Prism. Statistical significance was calculated by two‐tailed Student's *t* test unless specified otherwise in the figure legend. Data are expressed as mean ± SD. A *p*‐value < 0.05 in biological experiments or FDR < 0.05 in proteomics data analysis was considered statistically significant.

## 3 RESULTS

### 3.1 Characterization of serum sEV isolated by different methods

**FIGURE 1:**
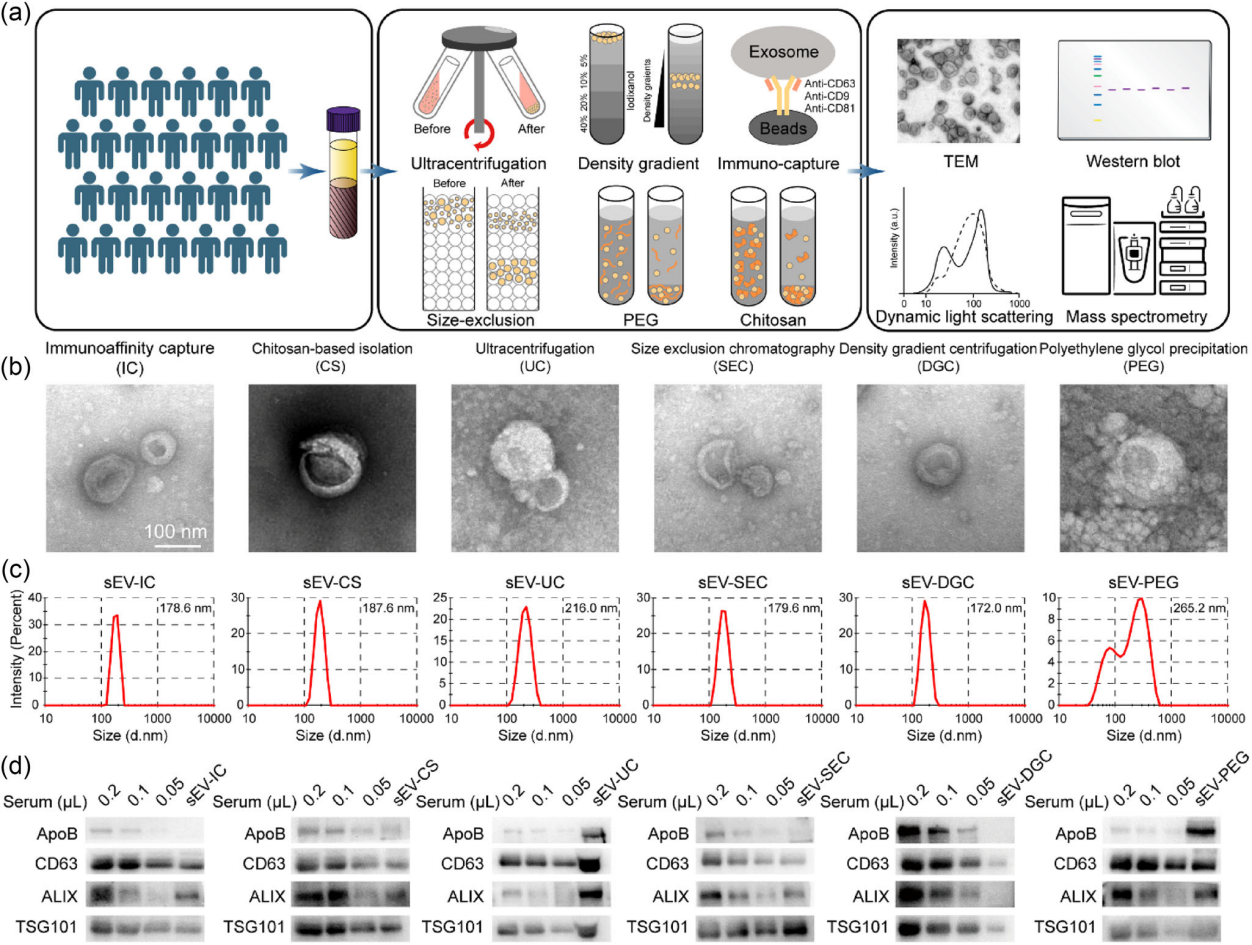
Characterization of sEV separated by distinct methods. (a) Workflow of evaluating sEV separated by IC, CS, UC, SEC, DGC and PEG. (b–d) Evaluation of sEV separated by distinct methods using TEM (b), dynamic light scattering (c), and western blotting (d). In the western blotting analysis, serum (0.2, 0.1 and 0.05 μL) was used as a positive control. sEV from 200 μL of serum separated by IC, CS, UC, SEC, DGC and PEG were loaded (right panel).

sEV is considered to have promising potential to be used as clinical therapeutic and disease biomarker. There are many recognized classic isolation methods, and a considerable number of novel methods under development. To compare the efficiency of sEV separation methods, serum sEV were isolated with UC, GDC, IC, SEC, PEG, and CS, and subjected to multiple analysis, including TEM, western blot, dynamic light scattering, and mass spectrometry (Figure 1a). The negative stain TEM and dynamic light scattering analysis revealed that sEV isolated by the distinct methods exhibited the cup‐shaped morphology (Figure 1b) with a diameter of 30–200 nm (Figure 1c). PEG isolated a larger population of spherical particles with a diameter of 20–30 nm, presumably to be lipoproteins, compared to other methods. UC also enriched some non‐sEV small particles, in addition to sEV. In contrast, IC, CS, SEC, and GDC mainly enriched sEV with few non‐sEV particles. Furthermore, western blotting demonstrated that sEV separated by distinct methods exhibited the expression of sEV markers (CD63, Alix, TSG101) (Figure 1d). PEG precipitated more lipoprotein ApoB compared to other methods. sEV isolated by UC showed high abundant sEV marker proteins, accompanied by relatively high levels of ApoB.

### 3.2 Proteomic analysis of sEV isolated by the distinct methods

**FIGURE 2:**
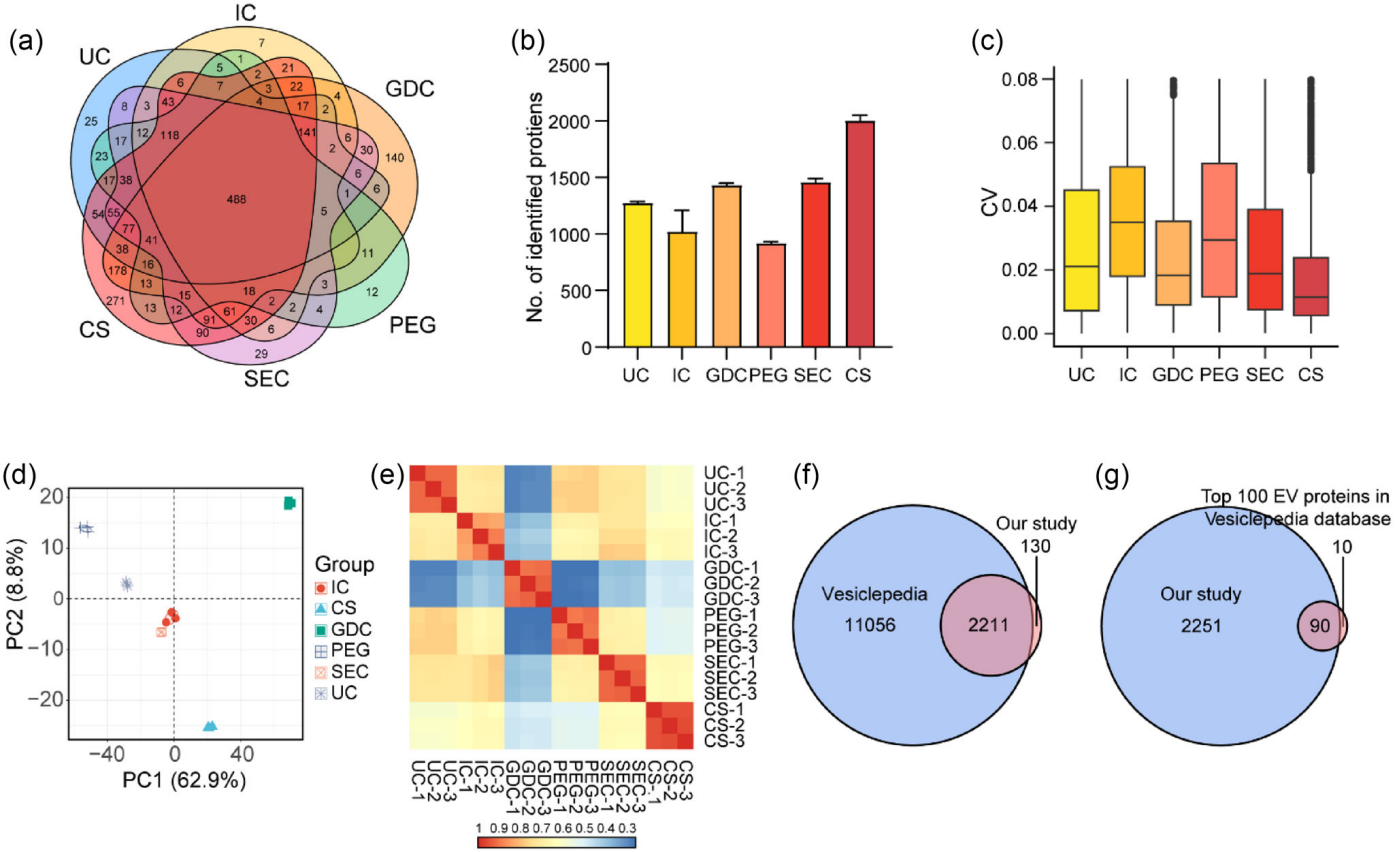
Proteome analysis of serum sEV separated by distinct methods. (a) Venn diagram of identified proteins in sEV separated by UC, GDC, PEG, IC, SEC, and CS. (b–d) Number (b), CV (c), and PLS‐DA plot (d) of identified proteins in sEV separated by each method. (e) Pearson correlation of identified vesicular proteins. (f&g) Venn diagram of identified vesicular proteins in our study and all proteins (f), or top 100 reported proteins (g) in the Vesiclepedia database.

Multiple proteins in sEV exert powerful efficacy in distinguishing cancerous and noncancerous patients. In this study, serum sEV was further evaluated by proteomics to explore whether the distinct methods affected sEV proteomic characteristics. The non‐labelled quantitative proteomics method based on DIA was applied. A total of 2340 proteins were identified, of which 1274, 1022, 1435, 920, 1460 and 2003 proteins, including 488 in common were found in sEV isolated from pooled serum using UC, IC, GDC, PEG, SEC and CS, respectively (Figuere 2a,b, Table S2). The reproducibility of the methods was further determined, revealing that CS had the lowest coefficient variation, followed by GDC, SEC, UC, PEG and IC (Figure 2c). Partial least squares discrimination analysis (PLS‐DA) of these identified proteins exhibited a clear separation between sEV separated by the distinct methods (Figure 2d). The sample correlation heatmap showed that sEV formed distinct clusters, suggesting that the dissimilarities among the samples were primarily driven by methodological variations rather than intrinsic differences (Figure 2e). To comprehensively evaluate the sEV separation methods, we compared all the identified vesicular proteins in our study with the Vesiclepedia database, one of the most commonly used and influential sEV databases (Kalra et al., 2012). The vast majority (95%) of proteins identified in our study were also present in this database, while 130 proteins have not yet been documented (Figure 2f). Of the top 100 mos t reported proteins in Vesiclepedia, 90 were found in our study, demonstrating the enrichment of known sEV cargo molecules (Figure 2g). Collectively, sEV released by the individual methods exhibited distinct protein profiles, and CS enriched most sEV proteins with lowest CV.

### 3.3 Evaluation of the distinct methods for sEV enrichment

**FIGURE 3:**
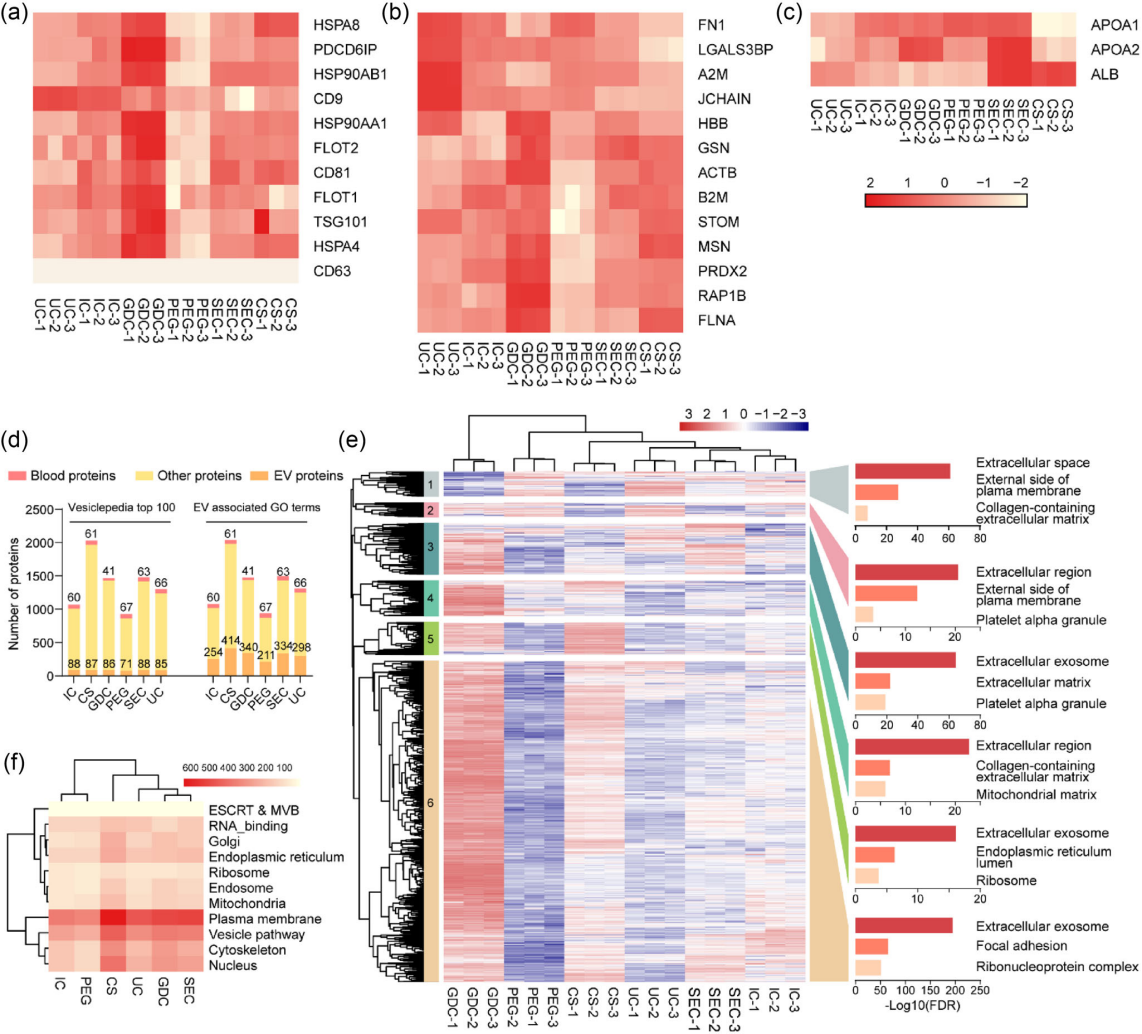
Comparative proteome analysis of sEV separated by distinct methods. (a–c) Levels of 11 conventional sEV protein markers (a), 13 newly defined sEV markers (b), and non‐sEV proteins (c) across sEV separated by distinct methods. (d) The comparison of identified proteins categorized as “blood proteins”, “Vesiclepedia top 100”, and “EV associated GO terms” in sEV separated by distinct methods. (e) HCA and GO enrichment analysis of the clustered vesicular proteins. (f) The comparison of identified proteins categorized for diverse cellular compartments.

To evaluate the purification of sEV isolated by the distinct methods, we investigated the levels of sEV markers. The expression of conventional sEV markers (Macias et al., 2018; Marrero et al., 2009) was highest in GDC, followed by CS, IC and SEC (Figure 3a). CD63 was absent in the serum sEV isolated by all the methods (Figure 3a), consistent with the result that CD63 is rarely detected in sEV from biofluids of either human or mouse origin (Hoshino et al., 2020). The levels of 13 novel common sEV markers (Hoshino et al., 2020) were highest in GDC, followed by CS, SEC and UC (Figure 3b). We also examined the presence of non‐sEV proteins, as indicated in the minimal information for studies of extracellular vesicles 2018 (MISEV2018) guideline (Thery et al., 2018), and lowest levels of APOA1/2 in sEV isolated by CS and ALB in sEV isolated by GDC were observed (Figure 3c). We further compared the number of identified sEV proteins annotated as Vesiclepedia top100 and EV proteins (Veerman et al., 2021). All the methods, except for PEG, isolated almost equal amounts of sEV proteins from Vesiclepedia top 100 list, and CS enriched most sEV proteins involved in EV‐related GO terms, followed by GDC and SEC. GDC isolated the least blood proteins, followed by IC and CS (Figure 3d).

The variation in sEV protein types was further determined using GO analysis. Hierarchical clustering analysis (HCA) with GO enrichment analysis were performed (Figure 3e), revealing that all methods mainly enriched for extracellular component as indicated by terms such as ‘Extracellular space’, ‘Extracellular region’, and ‘Extracellular exosome’ (Figure 3e). Cluster 3, 5 and 6 were mainly annotated as ‘Extracellular exosome’, and cluster 3 characterized by EV proteins such as ICAM1, integrins and RAB proteins RAB14, was mainly enriched by GDC, UC and SEC. Cluster 5 mainly driven by RAB1A and ESCRT III protein CHMP5, was enriched by GDC and CS. The largest cluster 6 characterized by EV proteins such as RABs, CD81, TSG101, FLOTs, integrins, CHMPs, ESCRT III associated protein VPS4B and RAB proteins, was mainly enriched by GDC, CS, IC and SEC, but barely enriched by PEG (Figure 3e).

Furthermore, the classification of identified sEV proteins based on 11 different cellular compartments and cell machineries was assessed. MV and exosome formation (Plasma membrane and Endosome, ESCRT and MVBs, especially Plasma membrane), and intracellular vesicle transport (Cytoskeleton and Vesicle Transport) was enriched mostly by CS, followed by SEC and GDC (Figure 3f). Collectively, these data suggest that CS enriches sEV with high purification, characterized by the identification of most sEV proteins, less non‐sEV proteins and high levels of sEV markers. Moreover, CS is a simple, time‐saving and cost‐effective method, which is more optimal for sEV biomarker discovery.

### 3.4 Optimization of CS‐coated magnetic beads for sEV enrichment

**FIGURE 4:**
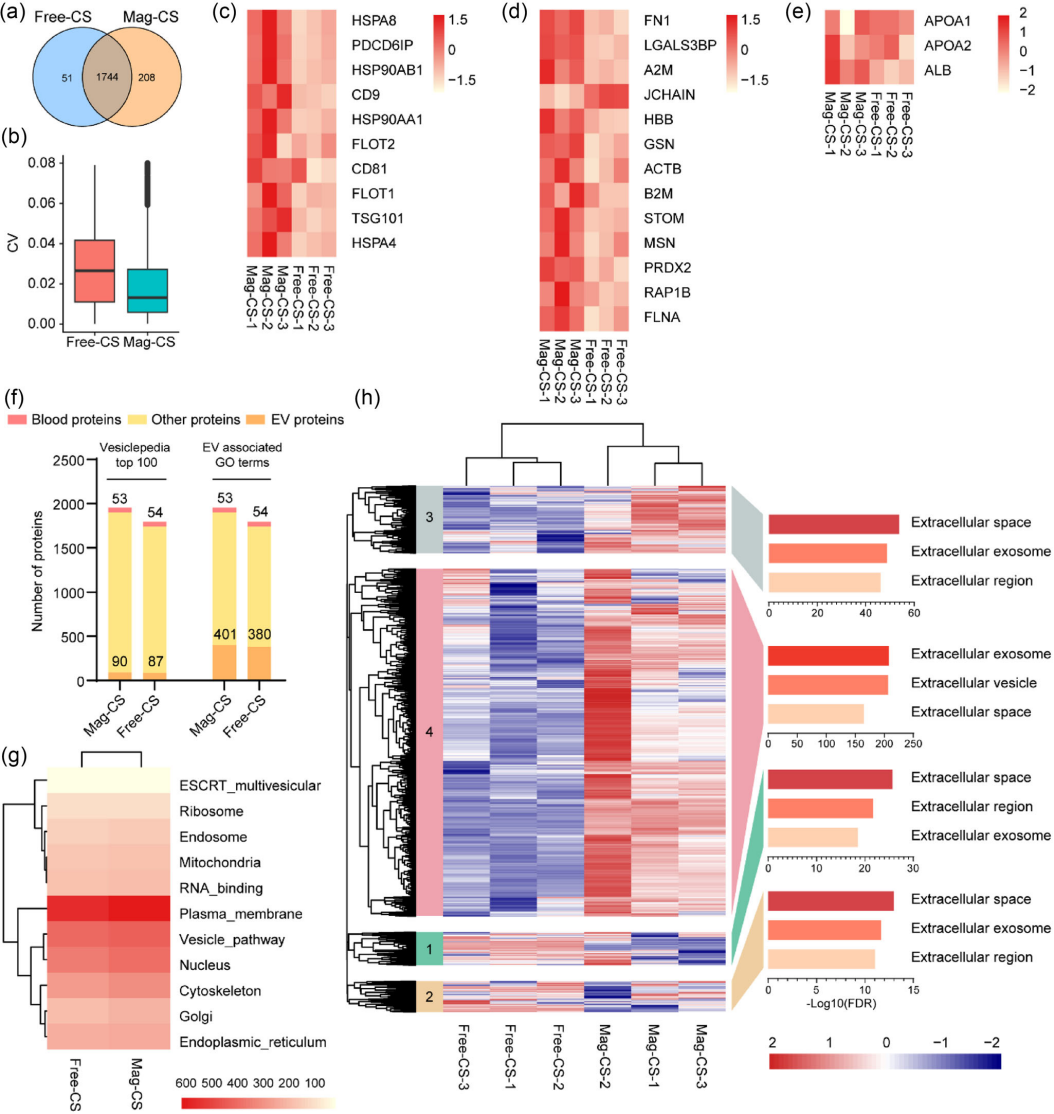
Comparative proteome analysis of sEV separated by free CS and Mag‐CS. (a) Venn diagram of identified vesicular proteins separated by free CS and Mag‐CS. (b) CV of identified vesicular proteins separated by free‐CS and Mag‐CS. (c–e) Levels of 11 conventional sEV protein markers (c), 13 newly defined sEV markers (d), and non‐sEV proteins (e) across sEV separated by free‐CS and Mag‐CS. (f) The comparison of identified proteins categorized as “blood proteins”, “Vesiclepedia top 100”, and “EV associated GO terms” in sEV separated by free CS and Mag‐CS. (g) HCA and GO enrichment analysis of the clustered vesicular proteins. (h) The comparison of identified proteins categorized for diverse cellular compartments.

Compared to precipitation‐centrifugation method, magnetic separation allows for the direct isolation of sEV from crude media under a magnetic field in a simple and high‐throughput way. Therefore, Mag‐CS were prepared, and sEV isolated by free CS and Mag‐CS were evaluated by proteomics. Most vesicular proteins were both identified in sEV enriched by free‐CS and Mag‐CS, with an additional 208 proteins exclusively identified in Mag‐CS (Figure 4a, Table S3). The coefficient variation of vesicular proteins enriched by Mag‐CS was slightly lower than that of vesicular proteins enriched by free CS (Figure 4b). Levels of conventional and novel sEV markers were significantly higher in Mag‐CS compared to free‐CS (**Figure** **4c,d**), and equal levels of non‐sEV proteins were identified (Figure 4e). More Vesiclepedia top 100 proteins and EV‐related proteins were enriched by Mag‐CS, accompanied by equal amounts of blood proteins (Figure 4f). HCA revealed that cluster 4 mainly annotated as ‘Extracellular exosome’ was enriched by Mag‐CS (Figure 4g). Identified vesicular proteins were predominantly categorized as plasma membrane, and exhibited no notable differences between the two methods (Figure 4h). Together, Mag‐CS exhibits more advantages in sEV isolation.

Nanoparticles of different sizes have been documented to exhibit distinct physical and chemical properties (Selmani et al., 2022). In this study, Fe_3_O_4_ nanoparticles with an average diameter of 20 nm (D20), 50 nm (D50), 100 nm (D100), 200 nm (D200), 300–500 nm (D3‐500) were employed to prepare Mag‐CS. Proteomic analysis revealed a specific expression pattern of vesicular proteins enriched by different Mag‐CS (Figure S1A, Table S4), especially a higher expression of sEV markers with lower expression of non‐sEV proteins in D3‐500 (Figure S1B–D). An equal level of proteins involved in Vesiclepedia top 100 proteins, EV associated GO terms and EV related cellular compartments were enriched by distinct Mag‐CS Figure S1E,F). Cluster 5, mainly annotated as ‘Extracellular exosome’, was enriched by D3‐500 (Figure S1g). Furthermore, the degree of deacetylation has been documented as an important parameter that determines many physiochemical and biological properties of CS (Freier et al., 2005; Prashanth et al., 2002), and different deacetylation degree (DD) of CS (ranging from 32% to 94%, termed as DD 1–5) were employed to prepare Mag‐CS. Proteomic analysis suggested a higher level of sEV markers and a lower level of non‐sEV proteins in DD‐5 (Figure S2A–D, Table S5), accompanied by an equal level of proteins involved in Vesiclepedia top 100 proteins, EV associated GO terms and EV related cellular compartments (Figure S2E,F). HCA revealed that cluster 2, mainly annotated as ‘Extracellular exosome’, was enriched by DD‐5 (Figure S2g). Collectively, CS with deacetylation degree of 93.9%, and Fe_3_O_4_ with a diameter of 300–500 nm were employed to prepare Mag‐CS for further sEV biomarker discovery for HBV associated HCC.

### 3.5 Proteomic analysis of sEV from HBV associated HCC patients

**FIGURE 5:**
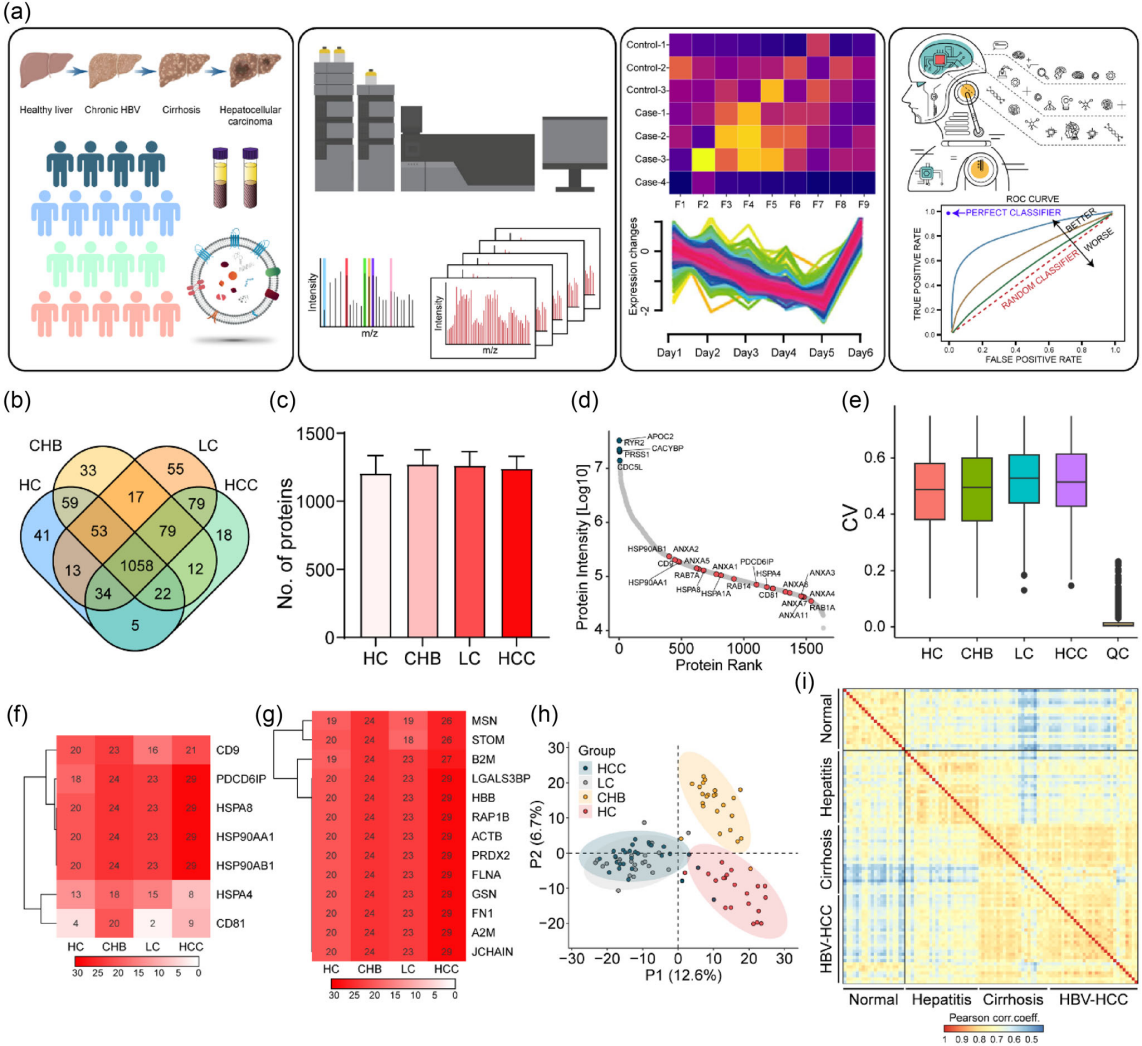
Proteome analysis of sEV derived from serum sample from HC, CHB, LC and HBV‐HCC. (a) Workflow of the proteome analysis of serum sEV. (b) Venn diagram of identified proteins in serum sEV from HC, CHB, LC and HBV‐HCC. An equal volume of serum (200 μL) was employed for sEV separation and proteomic analysis. (c) Number of identified proteins in sEV from different groups. (d) Rank plot of identified proteins in sEV. Commonly identified vesicular proteins are highlighted in red, and top 5 abundant vesicular proteins are highlighted in blue. (e) CV of identified vesicular proteins in sample cohorts and quality control samples. Pooled serum was used as quality control, that was included within the sample cohorts in proteomic analysis. (f&g) Positivity for conventional (f) and novel (g) sEV markers across different sEV. (h) PLS‐DA plot of identified proteins in serum sEV from HC, CHB, LC and HBV‐HCC. (i) Pearson correlation of identified vesicular proteins.

Emerging evidence suggests that HCC derived sEV can construct a fertile environment to support tumour progression, and sEV exhibit great potential to serve as novel noninvasive biomarkers. sEV derived from serum samples from healthy control (HC), CHB, HBV‐related liver cirrhosis (LC), and HBV‐HCC patients were isolated by Mag‐CS, and subjected to quantitative proteomics, functional analysis and machine learning (Figure 5a). A total of 1638 proteins were identified, of which 1285, 1333, 1388 and 1307 proteins, including 1058 in common were found in serum sEV from HC, CHB, LC and HBV‐HCC, respectively (Figure 5b,c). Although for certain vesicular proteins, concentrations increased with cancer stage (Peinado et al., 2012), we did not observe differences between these groups in the number of distinct sEV proteins. The MS signal intensities spanned four orders of magnitude, which reflected the identification and quantification of proteins with high and low abundance, including many know proteins (CD9, HSPs, RABs and ANXA proteins, etc) (Figure 5d). The coefficient variation of each group did not show any significant changes, and the coefficient variation of quality control sample was extremely low, indicating the high reproducibility (Figure 5e). The frequency of conventional sEV markers was further investigated, revealing the detection of seven of 11 conventional sEV markers (Figure 5f). All the novel sEV markers were present in most of the serum sEV from HC, CHB, LC and HBV‐HCC groups (Figure 5g). PLS‐DA analysis revealed significant proteomic differences between HC, CHB, and LC/HBV‐HCC, however, it could not separate LC from HBV‐HCC, indicating similar protein expression patterns between LC and HBV‐HCC (Figure 5h). Similarly, a correlation of the identified sEV proteins revealed that HC, CHB, and LC/HBV‐HCC samples showed higher correlation within than between groups (Figure 5i).

### 3.6 Serum sEV proteome differences between HC, CHB, LC and HBV‐HCC patients

**FIGURE 6:**
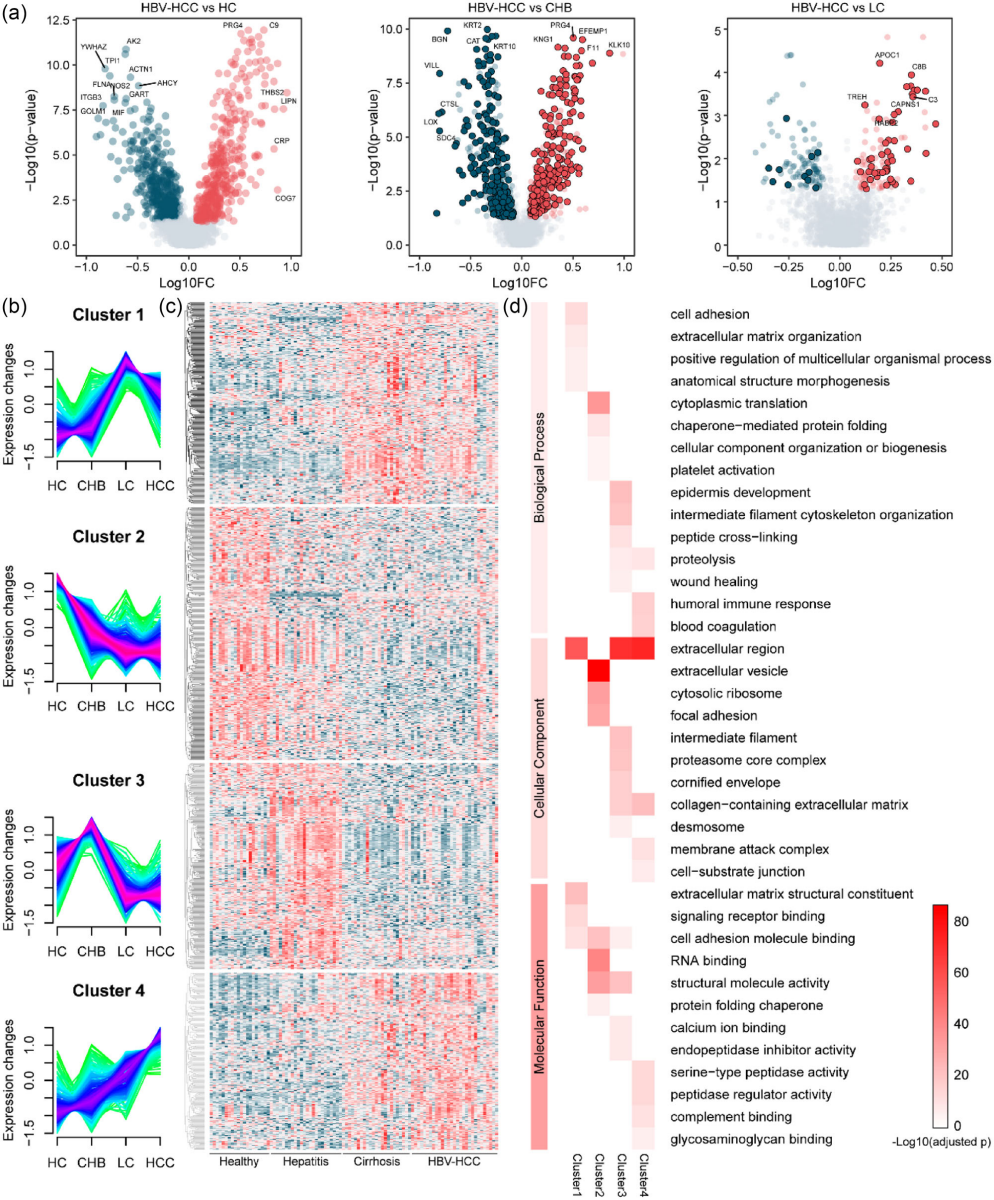
Differences and trajectories of vesicular proteins in the HBV‐HCC progression. (a) Volcano plot of the serum sEV proteomes in comparison of HBV‐HCC versus HC (left), CHB (middle), and LC (right). ‐log10 (*p*‐value) is plotted against log10 (FC, fold change) using cutoffs of fold change > 1.2, fold change < 0.83, and *p*‐value < 0.05 (Student's t‐test). Red, upregulation; blue, downregulation. Proteins continuously dysregulated in HBV‐HCC progression were highlighted. (b) Trajectories of serum sEV proteins that differentially altered in the HBV‐HCC progression (*p* < 0.05, one‐way ANOVA). Trajectories were clustered into four groups according to their expression patterns. (c&d) Hierarchical clustering map (c) and GO enrichment analysis (d) of the clustered vesicular proteins.

The alteration in serum sEV protein levels might enable the differentiation of HBV‐HCC from HC, CHB and LC. We compared the serum sEV proteomes between HBV‐HCC and HC, resulting in 833 sEV proteins with significantly altered levels (Figure 6a left; Table S6). The comparison of serum sEV proteomes between HBV‐HCC and CHB revealed 725 differentially expressed sEV proteins, with 218 continually increasing and 220 continually decreasing in the progression of HC‐CHB‐HBV‐HCC (Figure 6a middle; Table S7). Moreover, 224 sEV proteins were significantly expressed in HBV‐HCC compared to LC, of which 49 continually increased and 12 continually decreased in the progression of HC‐CHB‐LC‐HBV‐HCC (Figure 6a right; Table S8), which involved in complement binding and serine‐type endopeptidase activity (Figure S3A). The data indicate the rapid advances in HC‐CHB‐LC progression, and show that LC is closely related to HCC in the HBV‐HCC progression, consistent with the report that vast majority of liver cancer patients (80%–90%) suffer from cirrhosis (Konyn et al., 2021).

To understand the association between glycoprotein trajectories and the progression of HBV‐HCC, hierarchical clustering of the differentially expressed sEV proteins was performed, with which four cluster (I–IV) were generated (Figure 6**b,c**). sEV proteins in cluster I exhibited a significant increase, followed by a decrease, encompassing 266 proteins, which were enriched in extracellular matrix structural constituent and signalling receptor binding (Figure 6d). Proteins in cluster II were continually decreased (338 proteins), mainly involved in the RNA binding and structural molecule activity (Figure 6d). Cluster III exhibited partly similar trajectories as cluster I but decreasing levels towards the early state of HBV‐HCC (275 proteins), associated with structural molecule activity and calcium ion binding. Proteins in cluster IV were continually increased in the progression of HBV‐HCC (233 proteins), enriched in serine‐type peptidase activity and peptidase regulator activity (Figure 6d).

In addition to investigating differentially expressed sEV proteins in the progression of HBV‐HCC, we also mined sEV proteins exclusive to HBV‐HCC versus other groups (Figure S3b), mainly involved in the fibronectin binding (Figure S3c).

### 3.7 Analysis of specific DAMP molecules in serum sEV from HBV‐HCC

**FIGURE 7:**
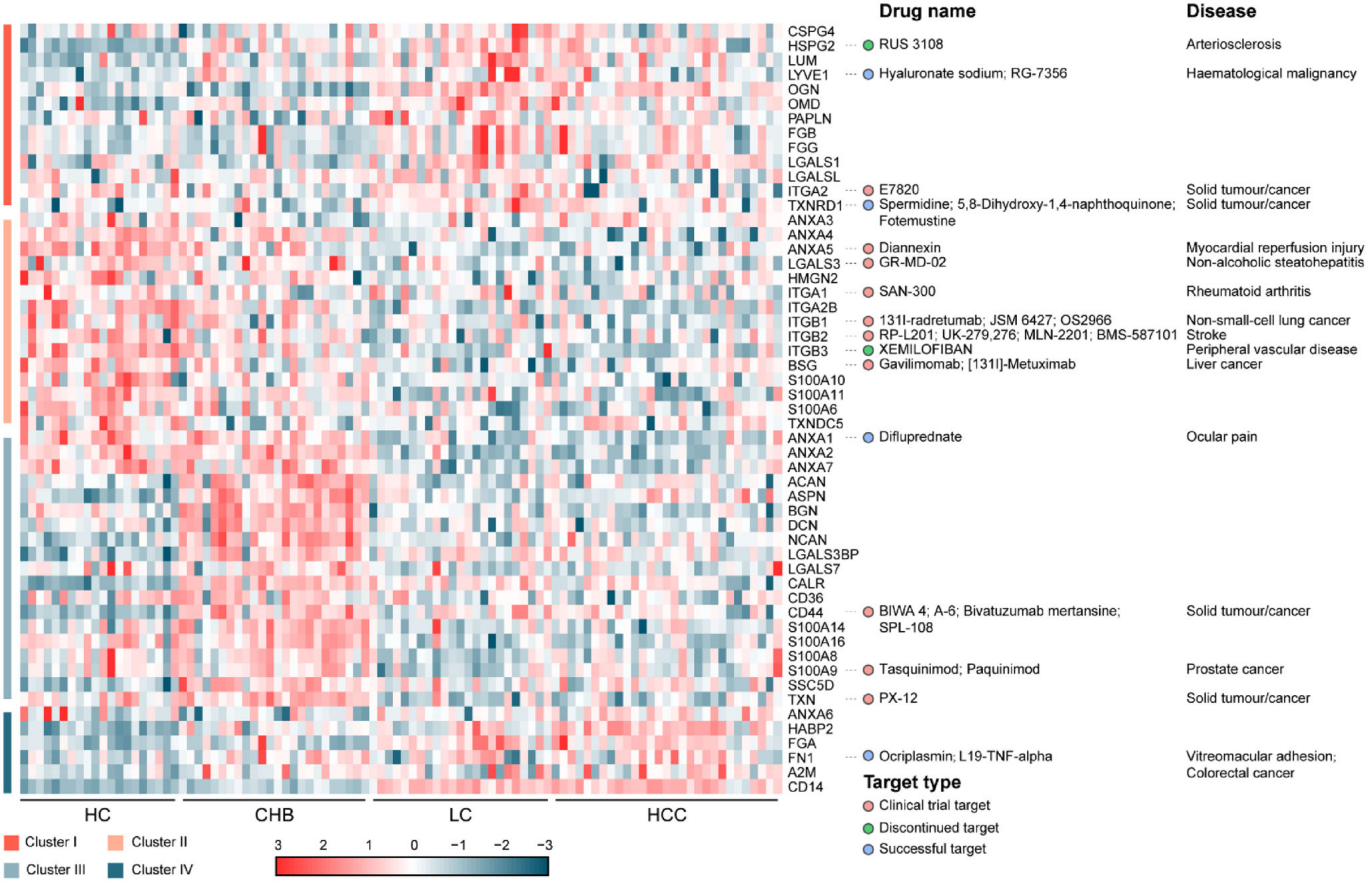
sEV DAMP molecules and associated potential therapeutic targets according to the Therapeutic Target Database. Fifty‐three sEV DAMPs were differentially altered in the HBV‐HCC progression, of which, 16 sEV DAMPs were identified as drug targets according to the Therapeutic Target Database (TTD). Target type, drug name, and disease were obtained from TTD.

sEV plays a pivotal role in immune regulation and cancer by intercellular communication and molecular transfer. We wonder whether specific molecules involved in inflammation and tumour immune, such as damage‐associated molecular pattern (DAMP) proteins (Hernandez et al., 2016), are packaged into sEV from HBV‐HCC serum. There were 53 sEV DAMPs differentially expressed in the HBV‐HCC progression (Figure 7). DAMPs in cluster I and III were mainly extracellular matrix (ECM) associated proteins, consistent with aberrant ECM deposition and remodelling during fibrosis (Wu et al., 2021), thus promoting an inflammatory immune response (McQuitty et al., 2020). A variety of integrins were mostly enriched in cluster II, indicated that the organ‐specific target of sEV might be altered in the progression of HBV‐HCC (Hoshino et al., 2015). We found six sEV DAMPs, including ANXA6, HABP2, FGA, FN1, A2M and CD14 in cluster IV were continually increased, which are effective receptors for pro‐inflammatory cytokines (A2M, CD14) (Hernandez et al., 2016; Raby et al., 2013) and pro‐inflammatory molecules (FN1, ANAXA6) (Hernandez et al., 2016; Raby et al., 2013). These data might help elaborate the immune and inflammatory role of sEV DAMPs in the progression of HBV‐HCC.

To better understand the potential of sEV DAMPs in therapeutic targets discovery for HBV‐HCC, these sEV DAMPs were cross referenced to drug targets using the Therapeutic Target Database (Wang et al., 2020), of which 16 sEV DAMPs were annotated as drug targets (Figure 7). Two sEV DAMPs were targeted by drugs for the treatment of liver diseases, including liver cancer and non‐alcoholic steatohepatitis. For example, BSG is a target of the drug [131I]‐Metuximab, which is undergoing clinical trial phase 2 for the treatment of liver cancer (Li et al., 2020). A total of eight and six DAMPs were targets by drugs for treating various cancer types and other diseases, respectively. For example, L19‐TNF‐alpha and Ocriplaimin that target FN1 (cluster IV), have been employed in treating colorectal cancer. Utilizing drugs that target sEV DAMPs might potentially provide an alternative option for combination therapy in HBV‐HCC cancer.

### 3.8 Vesicular protein biomarker signatures for detection of HBV‐HCC

**FIGURE 8:**
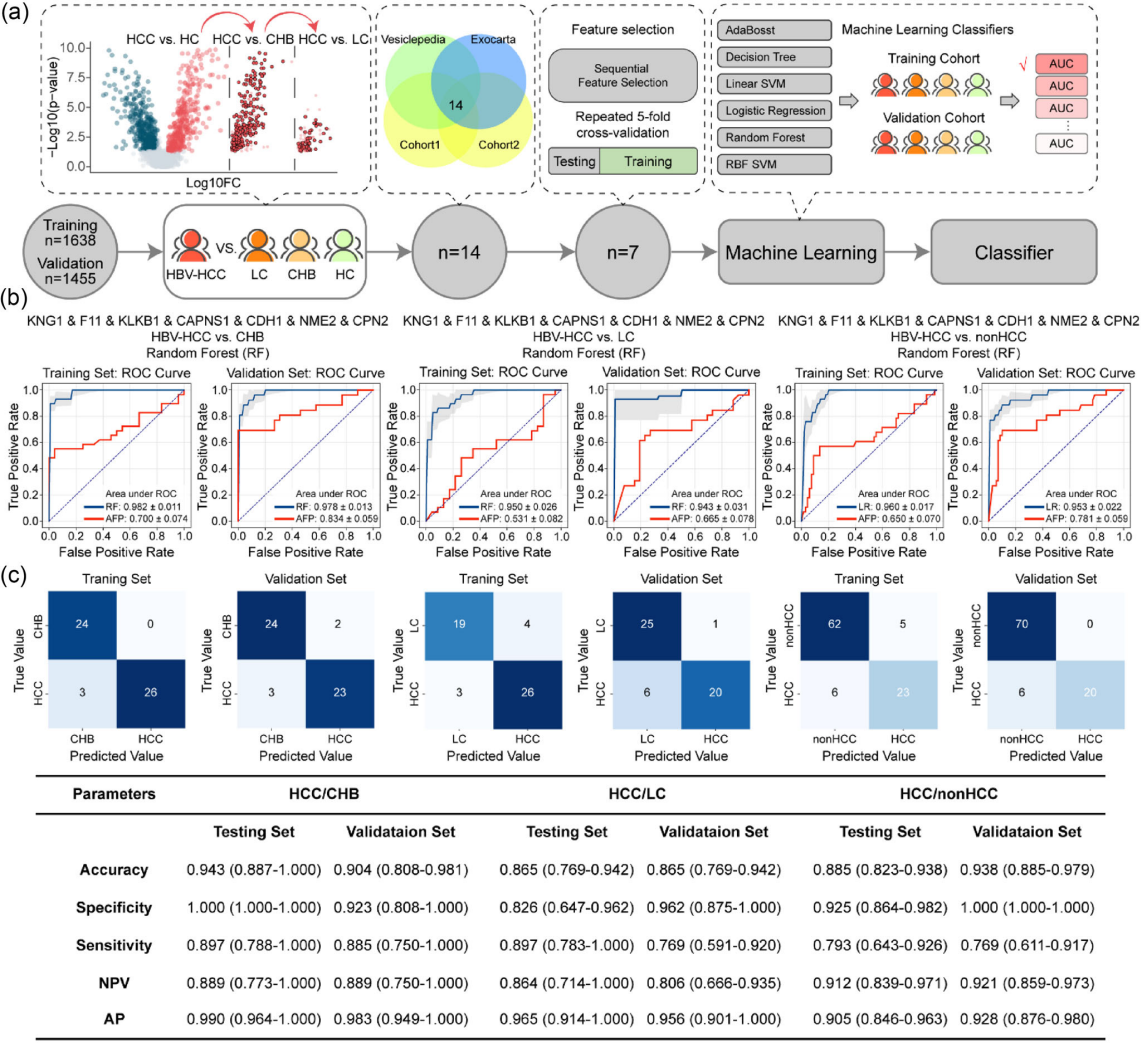
Serum vesicular protein signatures distinguishing HBV‐HCC using machine learning. (a) Workflow of feature selection and machine learning modelling. (b&c) ROC curve and confusion matrix performance of the vesicular protein panels in HBV‐HCC versus CHB, HBV‐HCC versus LC, and HBV‐HCC versus nonHCC using random forest model.

To validate vesicular proteins specific to HBV‐HCC progression, vesicular proteins were measured with DIA‐MS using an independent validation cohort of 96 patients (Table S1). A total of 1187 vesicular proteins were reproducibly detected in both cohorts (Figure S4A). We next explored whether comparing these specific proteins could distinguish HBV‐HCC from HC, CHB and LC. Machine learning was employed to identify potential combinations of biomarkers for classifying HBV‐HCC (Figure 8a). By training random forest models, a biomarker panel comprising KNG1, F11, KLKB1, CAPNS1, CDH1, CPN2, NME2, was identified by feature selection for liver disease detection (Figure S4b). We compared six advanced machine learning classifiers and selected a random forest machine model as the final classifier due to its excellent performance (Figure S5–S9; Table S9).

The panel performances were evaluated using AUCs and confusion matrix for pairwise comparison of HBV‐HCC, CHB, LC and non‐HCC (Figure 8b,c). Specifically, the AUC for detecting HBV‐HCC in CHB, LC and non‐HCC subjects are 0.978 (95% CI: 0.939–1.000), 0.943 (95% CI: 0.879–1.000) and 0.953 (95% CI: 0.910–0.995), accompanied by an AP of 0.983 (95% CI: 0.949–1.000), 0.956 (95% CI: 0.901–1.000) and 0.928 (95% CI: 0.876–0.980) in the validation set, respectively. Based on the confusion matrix, our vesicular biomarker panel achieved a sensitivity of 0.885 (95% CI: 0.750–1.000), 0.769 (95% CI: 0.591–0.920), 0.769 (95% CI: 0.611–0.917), and a specificity of 0.923 (95% CI: 0.808–1.000), 0.962 (95% CI: 0.875–1.000), 1.000 (95% CI: 1.000–1.000) in detecting HBV‐HCC in CHB, LC and non‐HCC, respectively. Notably, these findings are significantly higher than AFP alone (Figure 8b).

The SHAP bee swarm plots of three comparisons exhibited wide horizontal ranges on six of seven proteins, highlighting their significant contributions to the model's predictions. Among these proteins, even though CPN2 had small mean SHAP values (<0.01) in comparisons of HBV‐HCC versus HC and HBV‐HCC versus nonHCC (Figure S10). CPN2, a pleiotropic regulator of inflammation (Matthews et al., 2004), is also associated with the occurrence and development of multiple cancer types (Cui et al., 2016; Kaji et al., 2013).

## 4 DISCUSSION

Liquid biopsy tests show the potential for early cancer diagnosis, tumour classification, and therapeutic response monitoring. The sEV are commonly released more by tumour cells into bodily fluids and carry tumour‐specific content, representing an essential component of liquid biopsy test. It is important to separate sEV from bodily fluids in a simple, time‐saving and high‐throughput way, and to understand the content and types of sEV. To address these issues, we performed a thorough characterization and comparison of sEV isolated by distinct methods from human serum.

Upon cross‐referencing with Vesiclepedia databases, the majority of identified sEV proteins were annotated as vesicular proteins. Among these methods, GDC isolated sEV with the highest levels of sEV markers (**Figure** **3a,b**) and proteins related to ‘Extracellular exosome’ (Figure 3e). This aligns with the consensus that GDC is considered the gold‐standard method for achieving the highest‐purity sEV (Van Deun et al., 2014). Nevertheless, the utilization of GDC for sEV separation in clinical setting posed challenges, primarily due to the lengthy and tedious procedures, as well as low throughput (Coumans et al., 2017). CS stood out for the enrichment of high levels of sEV markers (**Figure** **3a,b**) and low levels of non‐sEV proteins, particularly APOA (Figure 3c), as well as proteins annotated as ‘Extracellular exosome’ and related terms (Figure 3d–e). Additionally, the largest number of sEV proteins were identified (Figure 2b). Notably, CS can be coated onto magnetic beads, providing a quite simple, time‐saving, and high‐throughput method for clinical testing. Mag‐CS were further prepared and compared, suggesting that CS (93.9% deacetylation degree) coated on magnetic beads (300–500 nm) performed better in sEV isolation. SEC and UC enriched high levels of sEV markers, moderate levels of proteins associated with extracellular exosome, and identified a moderate number of sEV proteins. Regarding experimental operations, SEC is quite simple, time‐saving, and high‐throughput. UC is the most commonly used methods, with the limitation of low‐throughput, time‐consuming and low purity yields. IC separated high levels of sEV markers, low levels of proteins related to extracellular exosome, and identified a small number of sEV proteins, probably due to the capture of sEV subpopulations with specific sEV membrane proteins. The use of IC for clinical testing is restricted due to its high cost of antibodies. The smallest number of sEV proteins was enriched by PEG with the lowest purity, indicating that high‐abundant non‐sEV proteins tended to mask the sEV protein. In addition, different levels of sEV markers might also indicate that different subtypes of extracellular vesicles were separated by the distinct methods. Collectively, CS, especially Mag‐CS might be the optimal method for serum sEV isolation in clinical trials. Mag‐CS was employed to isolate serum sEV for investigating the sEV hallmarker trajectory in the progression of HBV‐HCC.

Classical sEV biogenesis begins at the plasma membrane and matures into multivesicular body (MVB) formation. However, extensive research has reported that MVBs can also originate from various organelles, including mitochondria (Todkar et al., 2021), the nuclear envelope (Arya et al., 2022) and micronuclei (Yokoi et al., 2019). These non‐canonical sEV carries multiple mitochondrial proteins, including mtHSP70, Cyt C, TOM20 and OPA1, and nuclear content, including lamin A/C, histone H2A/B, importin and HSP70/90. In our study, these vesicular mitochondrial and nuclear proteins were enriched by CS (Table S2). Furthermore, studies demonstrated that these non‐canonical sEV exhibits some specific characteristics, such as enrichment of ceramide with negative charges (Arya et al., 2022; Trajkovic et al., 2008), which might facilitate the non‐canonical sEV separation by CS. These findings align with the enrichment of nucleus and mitochondria components by CS method (Figure 3f).

Interestingly, the HCA revealed four clusters of different longitudinal protein trajectories. The cluster I and III comprised proteins increasing from HC to LC/CHB patients and then decreasing in HCC patients. These proteins were enriched in extracellular matrix structural constituent and cell adhesion molecule binding, aligning with the deposition and remodelling of ECM in the LC patients (Crawford & Burt, 2012). Most of DAMPs in cluster I and III were also ECM, including HSPG, CSPG and LUM, termed ECM‐DAMPs. A number of ECMs, including HA, HSPG and CSPG, were documented to be transported to distant locations by attaching to the sEV membrane (Christianson et al., 2013; Schmidt et al., 2016), playing a critical role in the inflammation and immune (Hernandez et al., 2016; McQuitty et al., 2020). The dysregulation of these sEV proteins indicated that sEV, especially vesicular ECM, might be involved in the deposition and remodelling of ECM, as well as immune responses in liver diseases. Several drugs targeting ECM include Hyaluronate sodium, RUS3108 and RG‐7356. Hyaluronate sodium, a small molecular inhibitor of hyaluronic acid receptor (LYVE1), has been approved by the U.S. Food and Drug Administration for use in joint lubricant.

Proteins in cluster II were continually decreased in the progression of HBV‐HCC. These proteins were involved in cell adhesion molecule binding, which aligns with the observation that mostly DAMPs in cluster II were integrins (Figure 7). Integrins are transmembrane receptors responsible for anchoring cells to the ECM, and have involved in cell adhesion, migration, signal transduction. Integrin expression is markedly altered in liver diseases. For instance, livers from cirrhotic rats and patients with chronic hepatitis C and other end‐stage liver diseases exhibited highly expressed integrin αvβ6 compared to normal livers (Popov et al., 2008). While, hepatitis B virus X protein downregulated integrin α3β1 and α5, leading to a reduction in cell adhesion (Lara‐Pezzi et al., 2001). Integrins are commonly presented on sEV (Peinado et al., 2012), which were involved in several processes: horizontal transfer of integrins as vesicle cargo (Fedele et al., 2015), the preferential distribution of sEV (Peinado et al., 2012), or the recruitment of monocyte/ lymphocyte to the liver (Guo et al., 2019; Shetty et al., 2008). Collectively, integrins play versatile roles in HCC. Moreover, vesicular integrins were commonly elevated in various cancer types (Paolillo & Schinelli, 2017). However, several vesicular integrins were found to be decreased in the progression of HBV‐HCC, and the reduction in vesicular integrin β1 was validated by ELISA (Figure S12). The reason for the reduction of vesicular integrins during the progression of HBV‐HCC remains to be further elucidated using cellular biological and biochemistry techniques. Several drugs, including SAN‐300 targeting integrin α1, 131I‐radretumab, JSM6427 and OS2966 targeting integrin β1, RP‐L201 targeting integrin β2 in clinical trial phase, which is used to treat non‐small‐cell lung cancer and other diseases, hold the potential to serve as new therapeutic options for elimination of tumour microenvironments.

The cluster IV comprised proteins that were continuously increased from HC to HCC patients. These proteins were enriched in human immune response, blood coagulation and proteolysis. DAMPs in cluster IV included ANXA6, HABP2, FGA, FN1, A2M and CD14. A2M, relevant to human immune response, have been reported to be dysregulated in HCC (Kurokawa et al., 2003), and exhibited on the surface of sEV (Tronco et al., 2020). Alpha‐2‐macroglobulin, A2M, a broad‐spectrum protease inhibitor, characterized by its broader functions in immune system, including the promotion of antigen uptake, processing and presentation by antigen presenting cells, enhancement of specific cytotoxic T lymphocytes responses (Bowers et al., 2009), deposition of cytokines and growth factor (Huang et al., 1984; Petersen & Moestrup, 1990). Furthermore, direct interactions between A2M and proteins of the lectin pathway of complement, including mannose‐binding protein (MBP), MBP‐associated serin protease (MASP), suggest a regulatory role of A2M in the complement system (Terai et al., 1995; Vandooren & Itoh, 2021). Among these DAMPs, the alternatively spliced fibronectin (FN1) Type III domain, FnIII‐1c, can stimulate the activation of a toll‐like receptor 4 (TLR4)/NF‐κB or TLR4/MD2/CD14 pathway and the subsequent release of inflammatory cytokines in fibroblasts, indicating a regulatory role of FN1 in the TLRs‐mediated immune system. Ocriplasmin, a synthetic form of the serine protease and plasmin targeting FN1, has been approved by the FDA for use in vitreomacular adhesion, and might provide a potential option for HBV‐HCC therapy.

There is an urgent need to detect HCC from CHB and LC as they are the main risk factor of HCC. As the most widely used biomarker for HCC, AFP has limited specificity and sensitivity (Bruix & Sherman, 2011; Marrero et al., 2009), and was also elevated in non‐HCC malignancies (Crawford & Burt, 2012; Cui et al., 2016). Although a number of potential biomarkers were identified for improving HCC diagnosis, the sensitivity and specificity remain unsatisfactory (Best et al., 2020). In this study, machine learning with a logistic regression model was applied to develop a seven vesicular proteins panel to distinguish HCC from CHB with an AUC of 0.978, HCC from LC with an AUC of 0.943, HCC from nonHCC with an AUC of 0.953, which was significantly higher than AFP alone (Figure 8b). This panel of seven vesicular proteins shows potential as a diagnostic biomarker in serum for HCC, but further studies are needed to validate its clinical value. Among these vesicular proteins, CDH1 (E‐cadherin; E‐cad) was elevated in HBV‐HCC in both cohort (Figure S4b). As a typical transmembrane protein, E‐cad plays an important role in maintaining cell adhesion and intercellular stability (van Roy, 2014). In various types of cancer, reduced expression or dysfunction of E‐cad result in a damage in adherens junctions and an increased capacity for tumour cell invasion and metastasis. Notably, E‐cadherin can be cleaved off the ectodomain and released in a soluble form (sE‐cad) (Tang et al., 2018). sE‐cad is abundantly released in sEV, and sE‐cad positive sEV is closely associated with malignant ascites formation and widespread peritoneal dissemination in vivo assays and clinical studies (Tang et al., 2018). Similarly, peptides of E‐cad identified in our study were all localized in the extracellular domain rather than transmembrane domain or intracellular domain (Figure S11). These findings suggest that the elevated vesicular E‐cad observed in HBV‐HCC progression might be sE‐cad, which could facilitate the diagnosis and prognosis of HBV‐HCC.

## AUTHOR CONTRIBUTIONS

Study design: Feng Guan, Zengqi Tan, Lin Cao, Yue Zhou and Shuai Lin. Experimental work: Lin Cao, Yue Zhou, Shuai Lin, Chunyan Yang, Zixuan Guan, Xiaofan Li, Shujie Yang, Tong Gao and Jiazhen Zhao. Data analysis: Lin Cao, Yue Zhou, Ning Fan and Yanan Song. Visualization: Lin Cao, Yue Zhou and Shuai Lin. Data interpretation: Lin Cao, Yue Zhou, and Shuai Lin. Project supervision: Feng Guan, Zengqi Tan, Xiang Li and Dongmin Li. Drafting manuscript: Feng Guan, Zengqi Tan, Lin Cao, Yue Zhou and Zhuo Li. Revising manuscript content and approving final version: all authors.

## CONFLICT OF INTEREST STATEMENT

The authors declare that they have no conflict of interests.

## Supporting information



Supplementary Information

Supplementary Information

Supplementary Information

Supplementary Information

Supplementary Information

Supplementary Information

Supplementary Information

Supplementary Information

Supplementary Information

Supplementary Information

Supplementary Information

## Data Availability

All data needed to evaluate the conclusions in the paper are present in the paper and/or the Supplementary Materials. Raw data files have been deposited at jPOSTrepo (Japan ProteOme Standard Repository) (Okuda et al., 2017) accessible via the URL (https://repository.jpostdb.org/preview/100747486465daa9ea3eb1c) with the access key 7407.
