# Supplementary material for: The trajectory of vesicular proteomic signatures from HBV‐HCC by chitosan‐magnetic bead‐based separation and DIA‐proteomic analysis
Source: J Extracell Vesicles. 2024 Aug 29;13(9):e12499. doi: 10.1002/jev2.12499 (PMC11359709; doi:10.1002/jev2.12499)
Supplement: Supplementary file 2 — Supplementary Information [file JEV2-13-e12499-s003.docx]

The trajectory of vesicular proteomic signatures from HBV-HCC by chitosan-magnetic bead-based separation and DIA-proteomic analysis

Lin Cao^1^†, Yue Zhou^2^†, Shuai Lin^3^†, Chunyan Yang^4^, Zixuan Guan^2^, Xiaofan Li^2^, Shujie Yang^2^, Tong Gao^2^, Jiazhen Zhao^2^, Ning Fan^2^, Yanan Song^2^, Dongmin Li^5^, Xiang Li^1^, Zhuo Li^2,6^, Feng Guan^2^*****, and Zengqi Tan^1^*****

**Affiliations**

^1^ Institute of Hematology, Provincial Key Laboratory of Biotechnology, School of Medicine, Northwest University, Xi'an, Shaanxi, 710069, P.R. China.

^2^ Key Laboratory of Resource Biology and Biotechnology in Western China, Ministry of Education, Provincial Key Laboratory of Biotechnology, College of Life Sciences, Northwest University, Xi'an, Shaanxi, 710069, P.R. China.

^3^ Department of Oncology, The Second Affiliated Hospital of Xi'an Jiaotong University, Xi'an, Shaanxi, 710069, China.

^4^ Institute of Basic and Translational Medicine, Xi’an Medical University, Xi’an, Shaanxi, 710021, China.

^5^ Department of Biochemistry and Molecular Biology, School of Basic Medical Sciences, Xi'an Jiaotong University Health Science Center, Xi'an, Shaanxi, 710069, P.R. China.

^6^ Department of Laboratory Medicine, The First Affiliated Hospital of Xi’an Medical University, Xi'an, Shaanxi, 710077, P.R. China.

**Correspondence should be addressed to**

Feng Guan (guanfeng@nwu.edu.cn), Zengqi Tan (zengqtan@nwu.edu.cn). Tel: +86-29-88303534. Mailing address: College of Life Sciences, Northwest University, 229 Taibai North Road, Xi’an, Shaanxi, 710069, China.

† These two authors contributed equally

***** Corresponding authors

**This supplementary file includes:**

Figs. S1 to S10

**Other supplementary files includes:**

Table S1 to S9


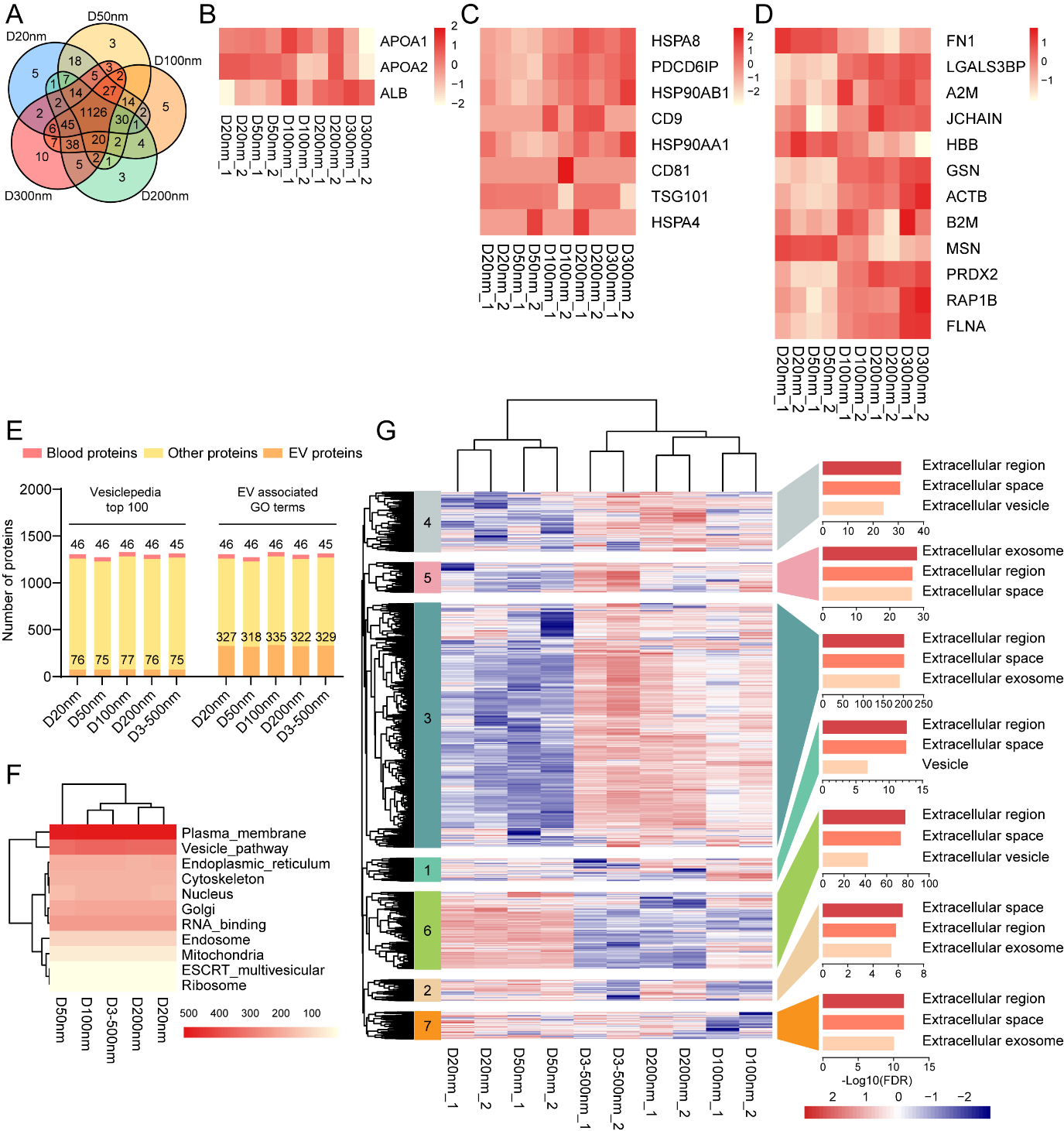


Fig. S1. Comparative proteome analysis of sEV separated by Mag-CS prepared with different size of Fe3O4 nanoparticles

(A) Venn diagram of identified vesicular proteins separated by Mag-CS prepared with different size of Fe3O4 nanoparticles. (B-D) Levels of 11 conventional sEV protein markers (B), 13 newly defined sEV markers (C), and non-sEV proteins (D) across sEV separated by Mag-CS prepared with different size of Fe3O4 nanoparticles. (E) The comparison of identified proteins categorized as blood proteins, “Vesiclepedia top 100”, and “EV associated GO terms” in sEV. (F) HCA and GO enrichment analysis of the clustered vesicular proteins. (F) The comparison of identified proteins categorized for diverse cellular compartments.


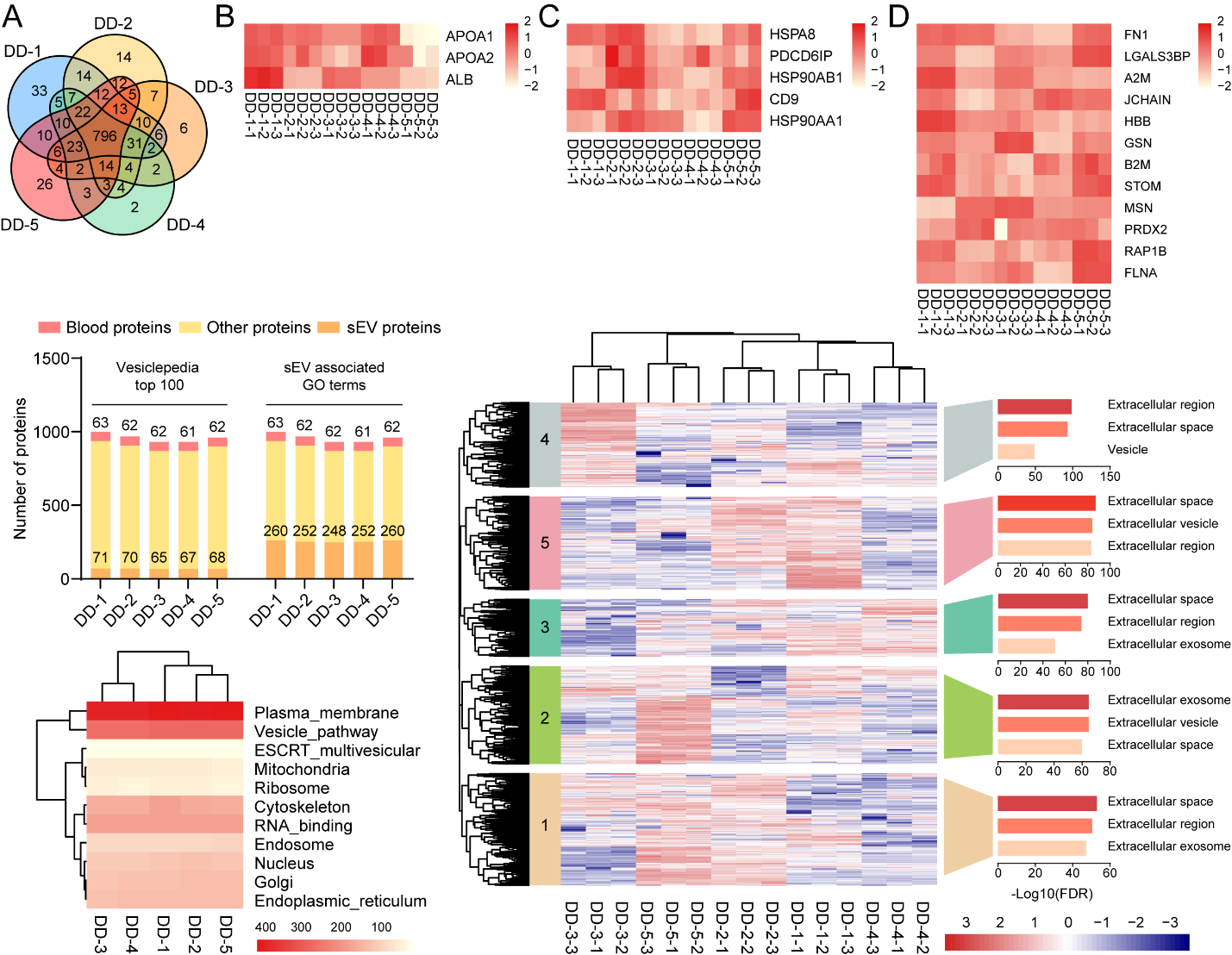


Fig. S2. Comparative proteome analysis of sEV separated by Mag-CS prepared with different deacetylation degree of CS.

(A) Venn diagram of identified vesicular proteins separated by Mag-CS prepared with different deacetylation degree of CS. Deacetylation degree of DD 1-5 is 32.7±0.02%, 76.5±0.01%, 86.1±0.01%, 57.4±0.03% and 93.9±0.02%, respectively. (B-D) Levels of 11 conventional sEV protein markers (B), 13 newly defined sEV markers (C), and non-sEV proteins (D) across sEV separated by Mag-CS prepared with different deacetylation degree of CS. (E) The comparison of identified proteins categorized as blood proteins, “Vesiclepedia top 100”, and “EV associated GO terms” in sEV. (F) HCA and GO enrichment analysis of the clustered vesicular proteins. (F) The comparison of identified proteins categorized for diverse cellular compartments.


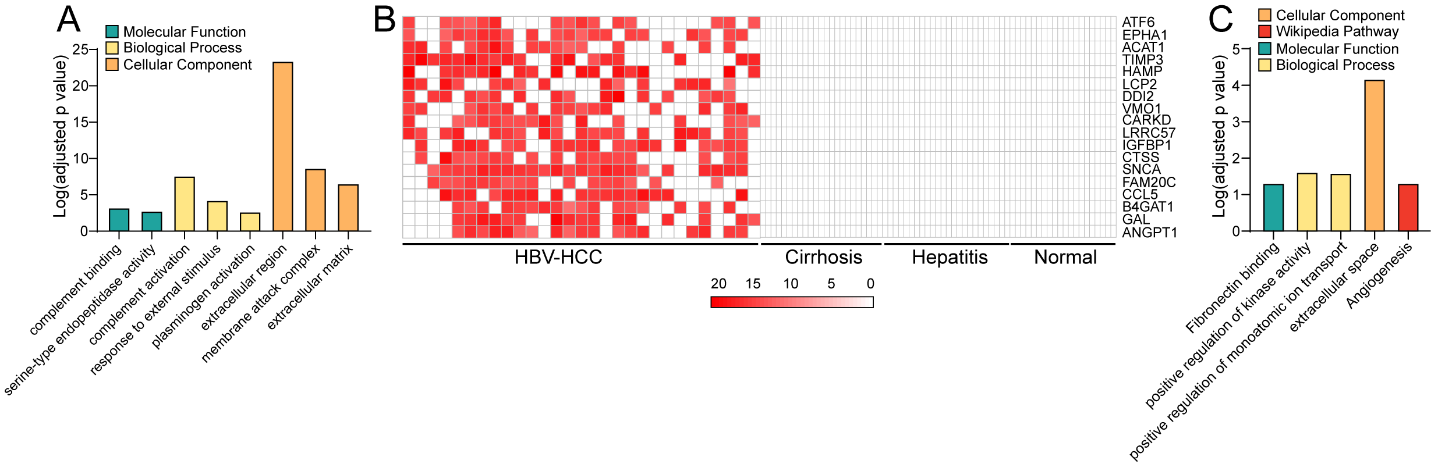


Fig. S3. Functional annotation of proteins dysregulated in HBV-HCC progression.

(A) GO enrichment analysis of vesicular proteins continuously up-/down-regulated in HBV-HCC progression. (B) Vesicular proteins exclusive to HBV-HCC versus other groups. (C) Functional enrichment analysis of vesicular proteins exclusive to HBV-HCC.


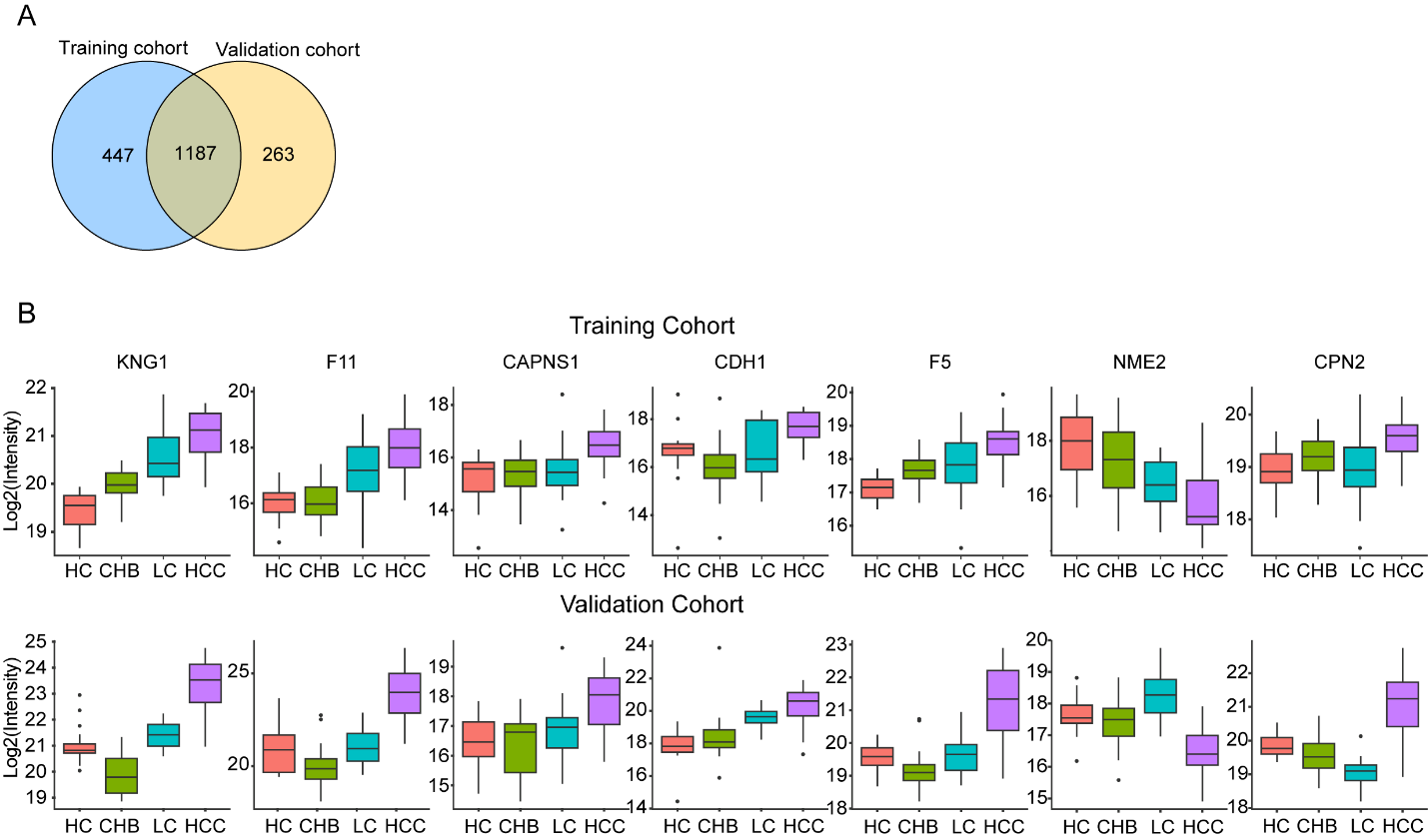


Fig. S4. Vesicular proteins identified in both training cohort and validation cohort. (A) Venn diagram of identified proteins in training cohort and validation cohort. (B) Expression of the multimarker panels in HC, CHB, LC and HBV-HCC patients.


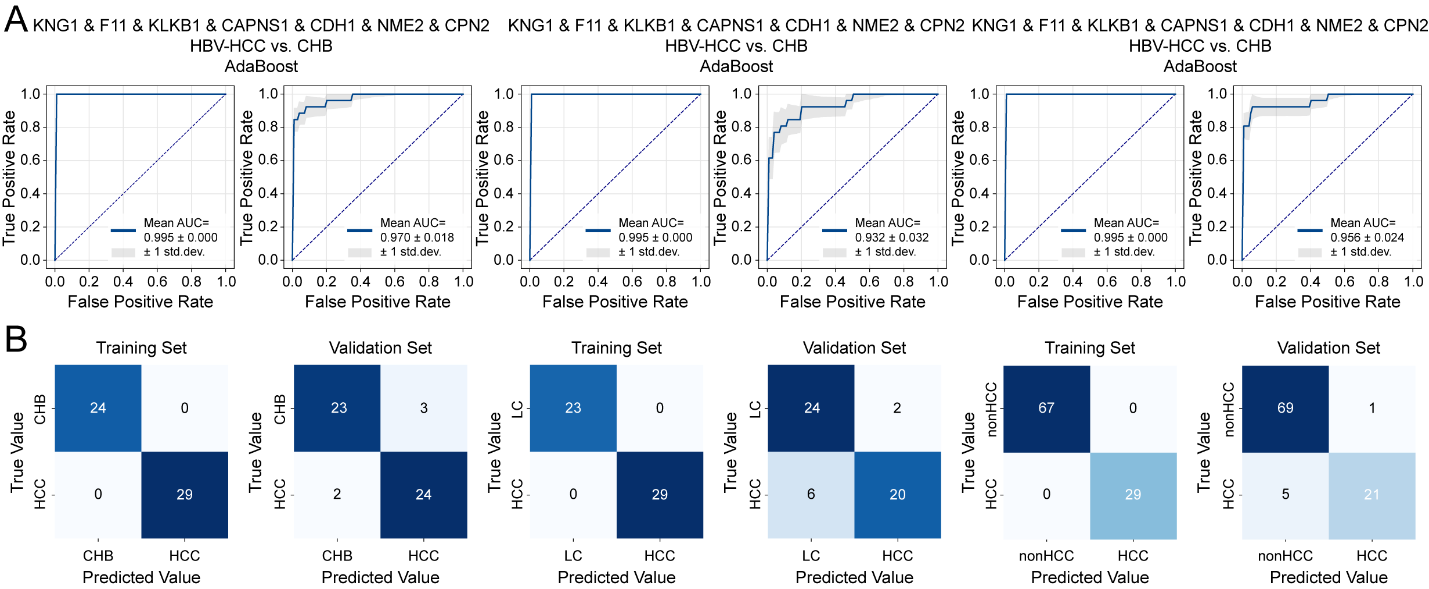


Fig. S5. Serum vesicular protein signatures distinguishing HBV-HCC using AdaBoost machine learning model.


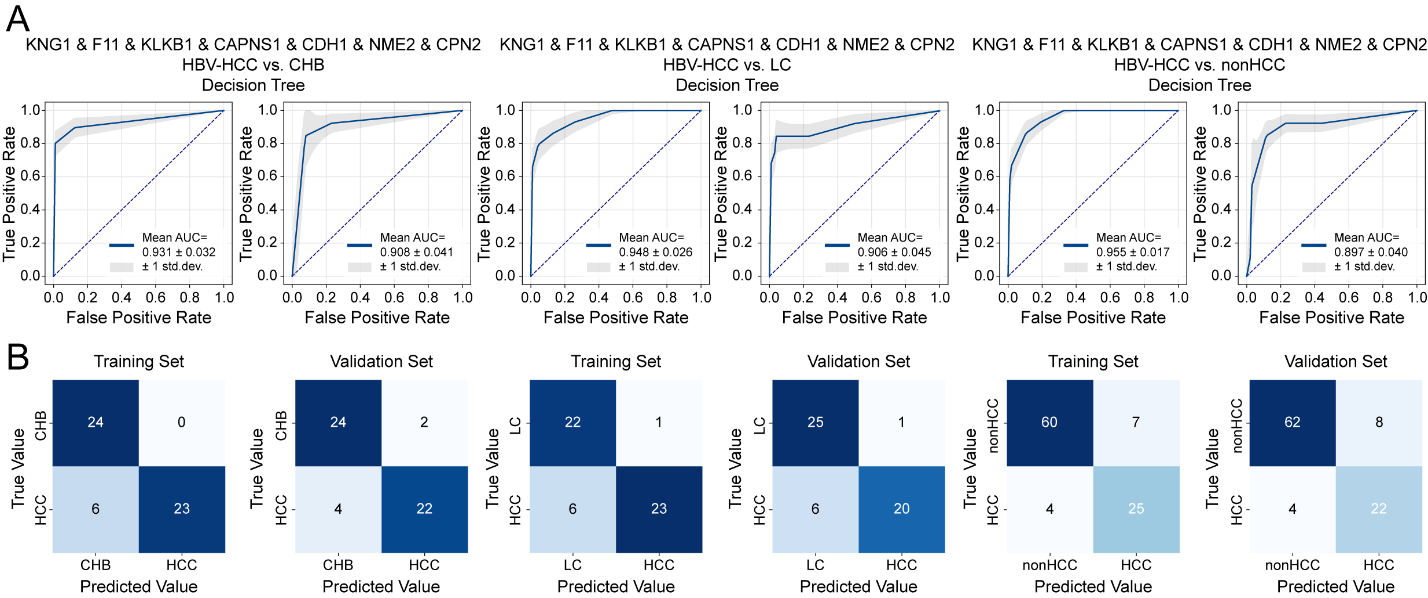


Fig. S6. Serum vesicular protein signatures distinguishing HBV-HCC using decision tree machine machine learning model.


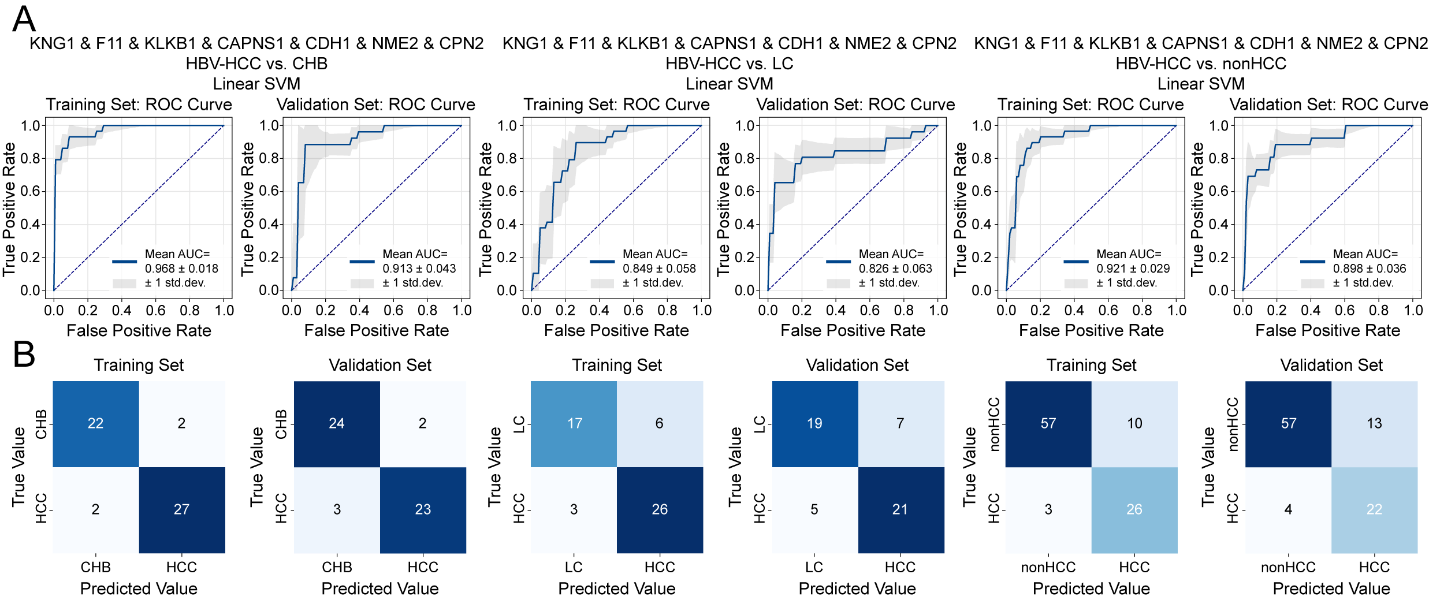


Fig. S7. Serum vesicular protein signatures distinguishing HBV-HCC using linear SVM machine learning model.


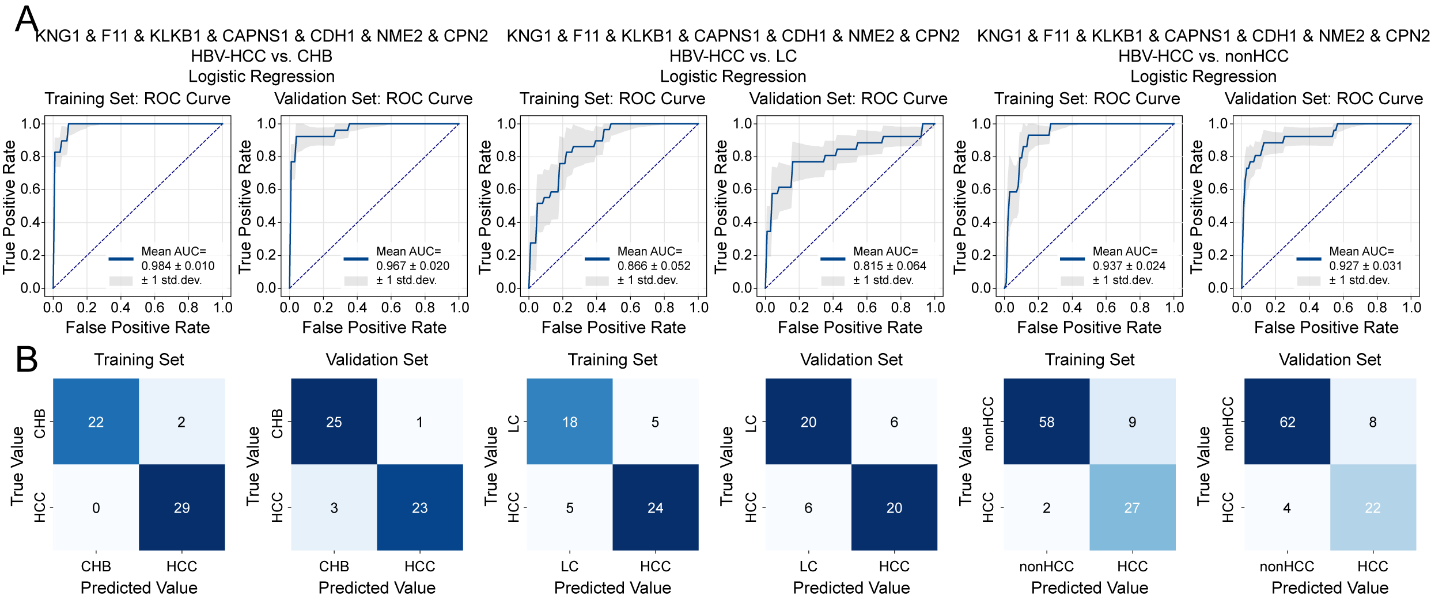


Fig. S8. Serum vesicular protein signatures distinguishing HBV-HCC using logistic regression machine learning model.


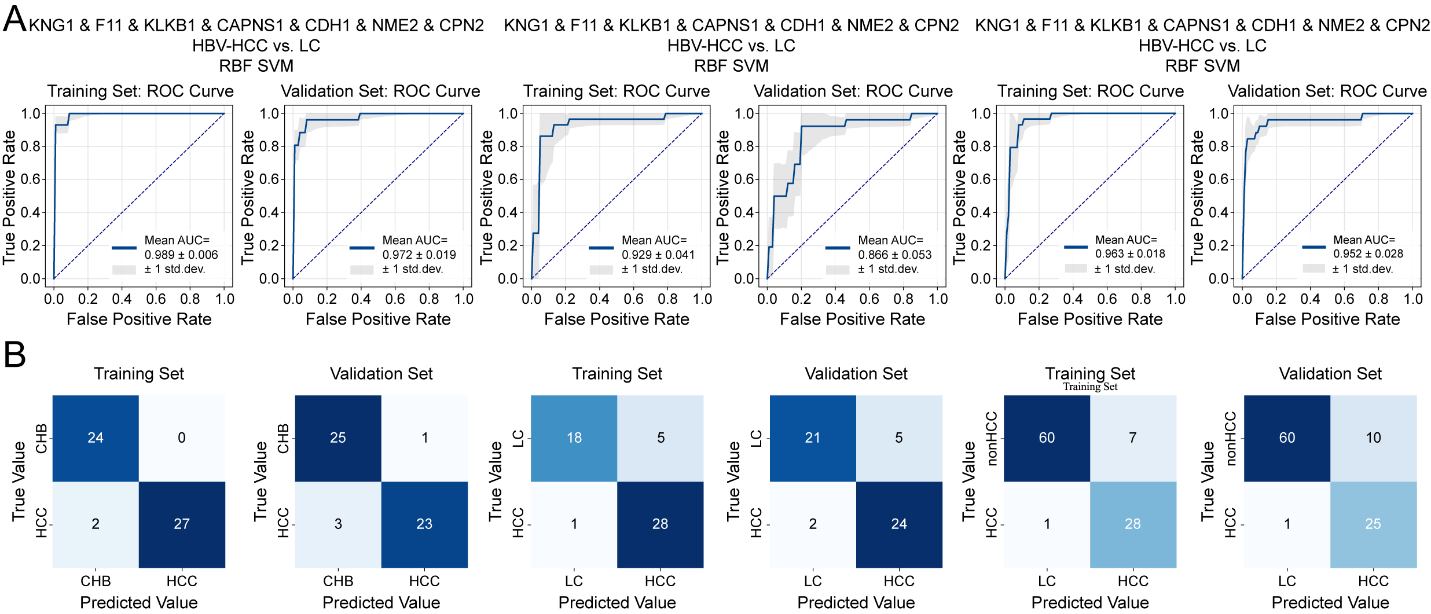


Fig. S9. Serum vesicular protein signatures distinguishing HBV-HCC using RBF SVM machine learning model.


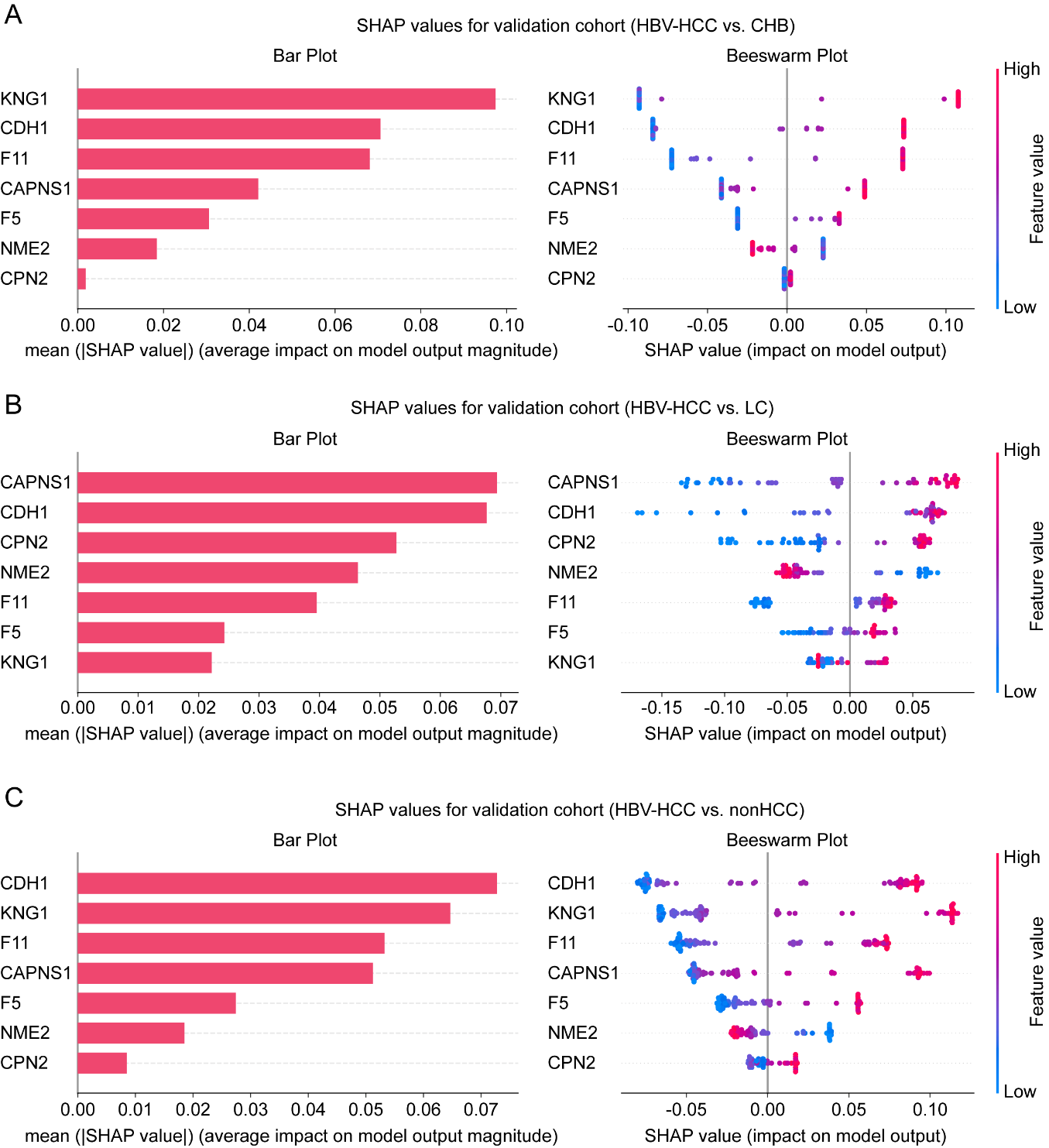


Fig. S10. Feature importance based on SHAP value. The mean absolute SHAP value (left) and the local explanation summary (right) of selected features in comparisons of HBV-HCC vs. CHB (A), HBV-HCC vs. LC (B), and HBV-HCC vs. non-HCC (C).


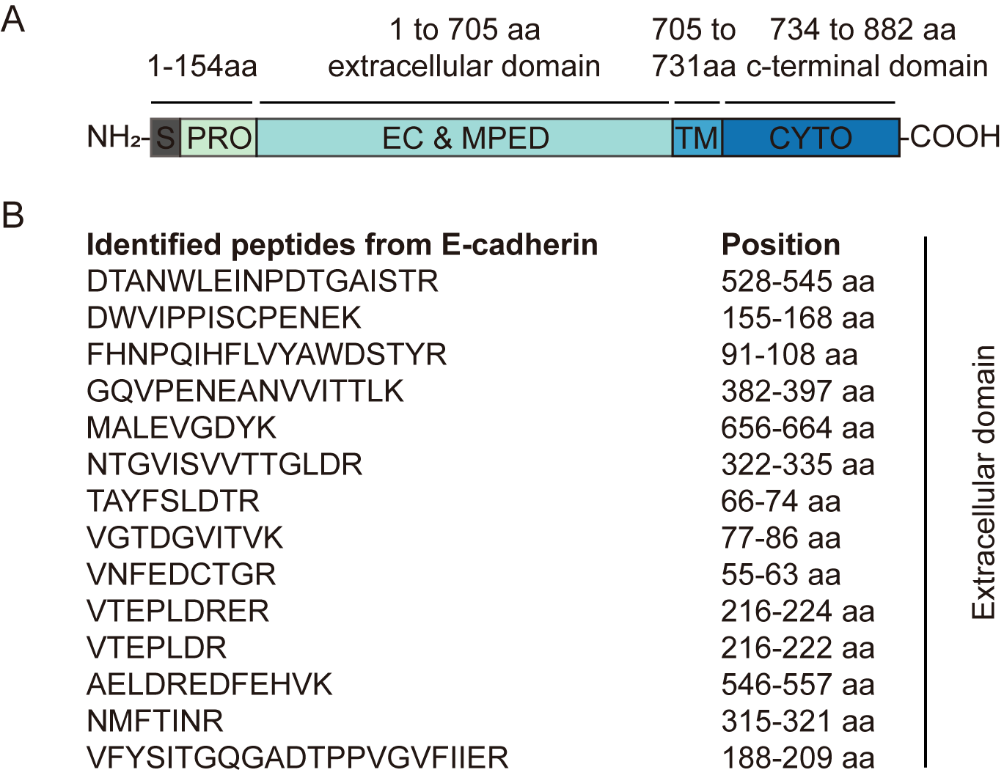


Fig. S11. Identified peptides from E-cadherin. (A) Schematic representation of different domains of E-cadherin. (B) Identified peptides from E-cadherin were all localized in the extracellular domain.

Other Supplementary Data.

Table S1. Characteristics of participants clinical data.

Table S2. Proteins identified in serum sEV using distinct methods.

Table S3. Proteins identified in serum sEV isolated by free CS and Mag-CS.

Table S4. Proteins identified in serum sEV isolated by Mag-CS prepared with different size of Fe_3_O_4_ nanoparticles.

Table S5. Proteins identified in serum sEV isolated by Mag-CS prepared with different deacetylation degree of CS.

Table S6. Dysregulated proteins in HBV-HCC versus HC.

Table S7. Dysregulated proteins in HBV-HCC versus CHB.

Table S8. Dysregulated proteins in HBV-HCC versus LC.

Table S9. Performance of the multi-marker panels in differentiating liver diseases.
